# Imaging Orientation of a Single Molecular Hierarchical Self-Assembled Sheet: The Combined Power of a Vibrational Sum Frequency Generation Microscopy and Neural Network

**DOI:** 10.1021/acs.jpcb.2c05876

**Published:** 2022-09-13

**Authors:** Jackson C. Wagner, Zishan Wu, Haoyuan Wang, Wei Xiong

**Affiliations:** † Department of Chemistry and Biochemistry, University of California San Diego, La Jolla, California 92093, United States; ‡ Materials Science and Engineering Program, University of California San Diego, La Jolla, California 92093, United States; § Department of Electrical and Computer Engineering, University of California San Diego, La Jolla, California 92093, United States

## Abstract

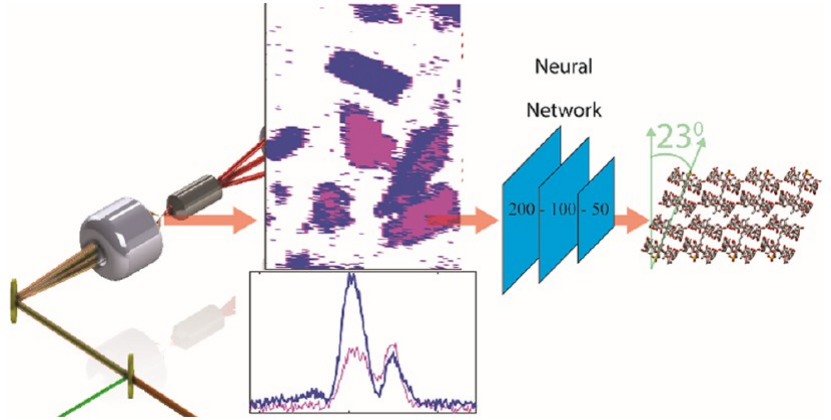

In this work, we determined the tilt angles of molecular units in hierarchical self-assembled materials on a single-sheet level, which were not available previously. This was achieved by developing a fast line-scanning vibrational sum frequency generation (VSFG) hyperspectral imaging technique in combination with neural network analysis. Rapid VSFG imaging enabled polarization resolved images on a single sheet level to be measured quickly, circumventing technical challenges due to long-term optical instability. The polarization resolved hyperspectral images were then used to extract the supramolecular tilt angle of a self-assembly through a set of spectra-tilt angle relationships which were solved through neural network analysis. This unique combination of both novel techniques offers a new pathway to resolve molecular level structural information on self-assembled materials. Understanding these properties can further drive self-assembly design from a bottom-up approach for applications in biomimetic and drug delivery research.

## Introduction

Molecular self-assemblies (MSAs) are a class of materials that spontaneously organize from individual molecular subunits into an ordered structure without templates or external guidance.^1−6^ MSAs can have larger architectures that maintain the high ordering and orientation of the smaller structures, known as hierarchical organization.^2,6−10^ In both natural and synthetic materials, even when formed from identical subunits, different hierarchical organization can lead to various material functions.^11,12^ For example, the diverse structures of natural collagen enable them to assume different tissue functions such as bone, skin, etc.^13^ In particular, bone possesses a 12-level hierarchical structure from collagen fibrils to the macroscopic fractal-like architectures, affording it both high stiffness and toughness, properties often considered mutually exclusive.^14^ Moreover, liquid crystal displays show different optical properties based on the orientation of the building blocks.^15^ Thus, it is feasible to design and manipulate materials functions through hierarchical organizations; however, to do so, it is necessary to understand and control the relative positions and orientations of the subunits within the MSAs.^16,17^

Specifically, the orientation of building blocks within a MSA is important to its functions. Applications can be found in chemistry, such as alkanethiol self-assembled monolayers, which are used as an active layer in molecular electronics. The tilting of the alkanethiol chains with respect to surface normal can open additional tunneling pathways and change interfacial dipole properties, altering the electron transport properties.^18−20^ In biomaterials, mechanical properties often depend on the hierarchical subunit orientation. For example, the longitudinal modulus of nacre is higher than the transverse modulus because of oriented tiles in the hierarchical structures.^21^ It has also been demonstrated that surface wettability is correlated with the tilting of subunits,^22^ which could further affect protein adsorption and cell adhesion.^23^

**Figure 1:**
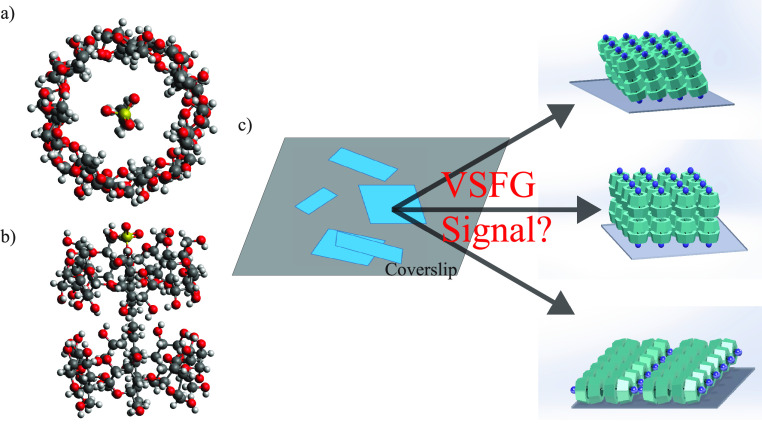
Structure of SDS@2β-CD. (a) Top down view of the SDS@2β-CD MSA subunits. (b) Side view of the subunits. (c) Microscopic formations of the SDS@2β-CD which hierarchically oriented to form rhombically shaped sheets. The orientation of the subunits relative to surface normal is unknown.

An interesting, recent development in MSAs is a lattice self-assembly composed of β-cyclodextrin (β-CD) and sodium dodecyl sulfate (SDS) in a 2:1 ratio, formed through intermolecular forces, especially hydrogen bonds.^11^ This MSA assumes a variety of morphologies depending on the concentration of SDS and β-CD in water. We will refer to this MSA as SDS@2β-CD herein. The primary subunit of the SDS@2β-CD self-assembly is the supramolecule comprised of two β-CD molecules penetrated by one SDS molecule (Figure 1a,b). These subunits form highly ordered and oriented rhombic sheets that can fold into larger mesoscopic architectures such as, lamella sheets, microtubules, rhombic dodecahedra, and micelles, among others.^11^ This MSA has drawn much attention because of the biomimetic nature of its mesoscopic architectures in addition to its broad application such as wastewater treatment,^24^ drug delivery,^25^ and optoelectronics.^26^ However, the structural details of the subunits, the most basic formation of the self-assembly that folds into all other higher order molecular architectures, are not fully understood.^11^ Through small-angle X-ray scattering (SAXS) the intersupramolecular distance was determined to be 1.52 nm, but open questions remain regarding the relative orientation of the subunits in SDS@2β-CD. Because molecular orientations often act as critical factors to MSA’s functions, it is pertinent to understand whether and how the subunits in the self-assembled sheets are tilted (Figure 1c), which could potentially indicate the van der Waals contact, the structural symmetry, or the materials macroscopic properties.^18,22,27,28^

In this work, we determined the orientation of the subunits in SDS@2β-CD through the development of a fast line-scanning vibrational sum-frequency generation (VSFG) microscopy in combination with neural network data analysis.^29,30^ Building on our previous efforts in ultrafast hyperspectral imaging techniques,^31,32^ this new technical advancement enabled imaging single SDS@2β-CD sheets hyperspectrally with eight different polarization combinations. Then, to reveal structural information, we applied a neural network method to solve a set of equations that relate the supramolecular tilt angle to the second order susceptibility of different polarizations. We found that the subunits were tilted relative to normal of the sheets by ∼23°. This provides insight into how to design future materials as well as offer details to what role hierarchical orientation played in MSAs. This structural knowledge is revealed through the combination of rapid acquisition of hyperspectral imaging and neural networks. Both are crucial to extract these parameters with the former minimizing long-term laser drift issues and the latter offering a route to solve a complex set of equations that were otherwise difficult to be solved.

## Method

### VSFG Line Scanning Microscope

The hyperspectral microscope was based on VSFG spectroscopy, a second-order nonlinear optical phenomenon. As an even-order nonlinear optical process, only noncentrosymmetric systems produce VSFG responses, such as the air/liquid, air/solid, and solid/liquid interfaces.^29,31−45^ Furthermore, VSFG is also sensitive to materials without inversion centers,^46−55^ many of which are MSAs, including collagen,^49,50^ amyloid fibers,^56^ artificial materials used in drug delivery,^57−59^ metal–organic frameworks,^60−62^ and piezoelectric crystals.^63^

A big challenge in using VSFG spectroscopy to probe MSAs is that most MSAs only form nano- to micrometer sizes domains, while the illumination area of VSFG spectroscopy is generally around 100 μm by 100 μm. Thus, traditional VSFG spectroscopy will probe multiple MSAs in an ensemble-averaged manner which does not accurately reflect the molecular structure of an individual MSA.^32^ The development of VSFG spectroscopy into a hyperspectral imaging technique^32,48−50,52,53,64−67^ overcame this challenge with 1 μm or submicrometer resolution being obtained, which offered a platform that could resolve multiple micrometer-sized MSAs individually.

Additionally, to retrieve molecular orientations, it was necessary to measure VSFG images with multiple polarization combinations.^49,50,65,68^ Though theoretically feasible, it was practically prohibited in our previous point-scanning VSFG microscope^31,32^ since it took nearly 4 h to scan a single 100 μm by 100 μm polarization resolved image and would take at least 30 h to collect all eight polarization combinations. The long acquisition time would introduce fluctuations in optomechanics and laser output, which further complicates data analysis.

To overcome these challenges, we hybridized the line-scanning technique with our existing VSFG microscope. A line-scanning method was first implemented in VSFG microscopy by the Ge and Potma groups using a photomultiplier tube as a detector,^49,50,64,65^ which required scanning the IR frequency to gain spectral information. The integration of line-scanning with a CCD detector reported here enabled simultaneous measurement of spectra of a vertical line, maximizing the information measured by the 2D detector.

**Figure 2:**
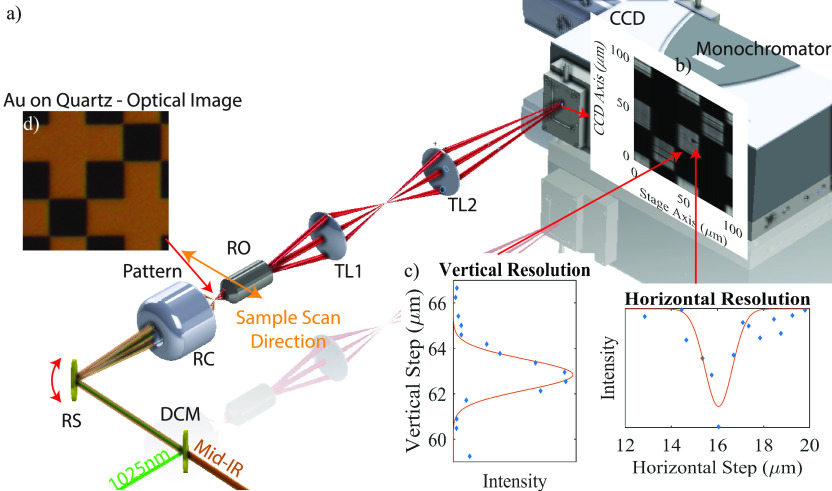
Line-scanning VSFG microscope. (a) Schematic of the setup: DCM, dichroic mirror; RS, resonant scanner; RC, reflective objective; RO, refractive objective; TL1, convex lens 1 in tube lens; TL2, convex lens 2 in tube lens. (b) VSFG image of quartz substrate target image. (c) Vertical and horizontal resolution generated by taking the derivative of the corresponding cut. The spectral resolution is 4 cm^–1^. (d) Optical image of gold target on quartz substrate in a similar area.

The VSFG line-scanning microscope is shown in Figure 2. Laser pulses for the microscope are provided by a 100 kHz Yb based cavity femtosecond laser (Light Conversion, Carbide) centered at 1025 nm. The output from the Carbide is used to pump an optical parametric amplifier (OPA) (Light Conversion, Orpheus-HP) centered at 3500 nm which covers the CH stretching vibration region of interest. The residual 1025 nm beam is used as the up-conversion and is first conditioned spectrally by directing it through a folded 4f pulse shaper. The frequency narrowed 1025 nm beam is then focused through an 8 μm spatial filter followed by a λ/2 waveplate. The mid-IR (MIR) light is steered toward a delay stage followed by a λ/2 waveplate and spatially overlapped with the up-conversion with a customized dichroic mirror that is transparent to MIR and reflective to near IR (NIR). In this way, the mid-IR and upconversion beams are combined collinearly. The overlapped beams are then guided to a 1D resonant scanner (EOPC) and focused onto the sample mounted to a 2D piezo stage (MadCity Labs) by a purely reflective 20× Schwarzschild objective (0.70 NA, PIKE Technologies Inc., PN 891-0001). The emitted nonlinear VSFG signal is collected by an infinity corrected, 20× refractive microscope objective (Zeiss, Fluar 0.75 NA, working distance = 0.6 mm) and passes through a linear polarizer (ThorLabs). The polarization resolved signals are projected into spectra using a Shamrock 500i spectrograph (Andor) coupled to a CCD (Newton, Andor).

To enable line-scanning, the resonant scanner operated at 325 Hz and steered the angle of the beam along the vertical axis. At the sample plane, the incoming beams were transformed into a vertical line of illumination which generated a line of VSFG signal that was relayed and magnified by a home-built tube lens to match the vertical dimension of the CCD. The signal was frequency resolved by a spectrograph horizontally (Figure 2a). Thus, the CCD measured the spectra dispersed along the horizontal axis and the spatial profile along vertical axis. 2D images were acquired by scanning the sample in the horizontal direction with an automated mechanical stage. This improvement decreased image acquisition times by 10, compared to the point-by-point microscope, to 20 min for a 100 μm by 100 μm image.

The VSFG images obtained from the line-scanning microscope captured the same geometric features of the optical image of plated gold patterns on quartz substrates (Figure 2a,b,d). Depending on the scan angle of the resonant mirror and magnification of the tube lens, we achieved a 100 μm vertical field of view. The vertical and horizontal resolutions were 1.2 and 1.6 μm, respectively (Figure 2c), and the total magnification was 66.

### 2B-CD@SDS Synthesis

B-CD sheets are synthesized by adding B-CD and SDS at a molar ratio of 2:1 in DI water until the percent concentration is 10% m/m. The suspension is then heated to clarity and cooled to room temperature overnight. CuCl_2_ is added with sheets fully forming approximately 3–5 days later. Isolated sheet samples with linear dimensions on the 10s of micrometer scale are produced by drop casting 5 μL of the sheet suspension onto a 15 mm × 15 mm × 0.170 mm microscope slide spinning at 10 000 rpm. The sheets are transparent, but the silhouette can be observed with a standard optical microscope.

### Neural Network Model

**Figure 3:**
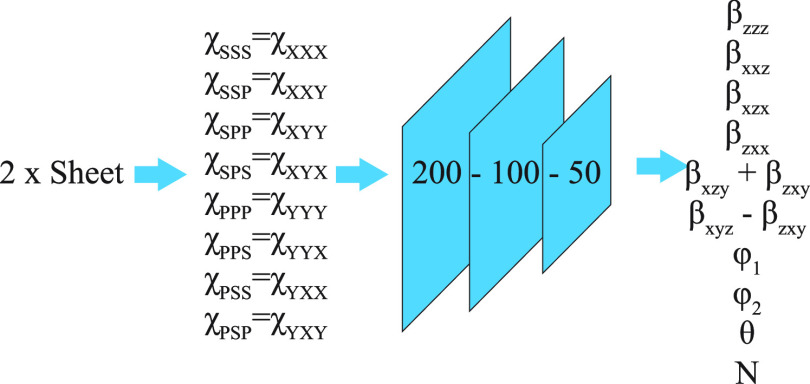
Schematic illustration of the neural network employed to extract orientation information on the SDS@2β-CD.

The polarization dependent VSFG spectra were analyzed using a neural network model to extract molecular tilt information. Keras in Python is employed to set up the neural network model. A layered neural network modified from Github repotsitory^69^ is built with a 200–100–50 node structure and a hyperbolic tangent activation function between layers (Figure 3). Training set with 100 000 by 27 values (see Supporting Information for details) was created by randomly generating angles and hyperpolarizabilities (used as training output, 100 000 by 11 values) and then supplying the values to the polarization-dependent equations (eqs 5–12 in Supporting Information) to calculate the corresponding susceptibilities (used as training input, 100 000 by 16 values) (Figure 3, Figure S13a). Similar to simple trigonometric function where θ = 0 and θ = π both satisfy sin(θ) = 0, one input vector (susceptibility values) in our model could also have multiple output vectors (different in-plane rotation, tilt angle, hyperpolarizabilities combinations) at the same time. Hence, the in-plane rotation angle is divided into [0, π) and [π, 2π) intervals and the tilt angle is divided into [0, π/2) and [π/2, π) intervals to differentiate these output vectors. Training is run with an epoch size of 1000 and a batch size of 100. Mean squared error of output vector is used to monitor the deviation of prediction from true values. During the training, only 90% of the data set is used for actual modeling and the rest 10% of the training data is separated out to testify whether the model can generalize to unseen data. In such a way one can mitigate overfitting, as seen in the small and close loss values obtained in both training and test processes (Figure S13b).

After validating that our model is capable of predicting the tilt angle, we supply the model with experimentally determined susceptibilities Figures S9 and S10, Table S1) to extract the tilt angle. Since there is no phase information in our measurement, we enumerate the sign of each susceptibility value (total of 2^16^ combinations) and provide all these as input vectors to our neural network.

## Results and Discussion

### Polarization Resolved Hyperspectral Imaging on SDS@2β-CD

To measure the tilt angle of subunits in SDS@2β-CD, we applied the line-scanning VSFG microscope to image single SDS@2β-CD sheets. Single SDS@2β-CD sheets, which can be visually seen under optical microscope with micrometer size, were carefully prepared (synthesis details in Supporting Information). We note that the single sheets are composed of multilayers of self-assembled materials instead of a monolayer, and its thickness is about 110 nm, determined by AFM height profiles (Figures S2 and S3). Via inspection with an optical microscope, we mitigate sheet overlapping, which would complicate analysis later as well as affect the image quality, by optimizing spin coating parameters (Figure S1).

**Figure 4:**
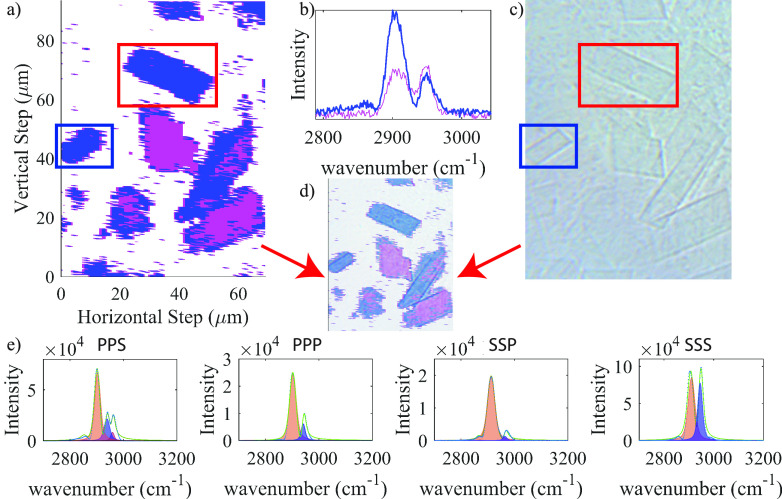
VSFG hyperspectral image and spectral analysis of the SDS@2β-CD. (a) Polarization resolved hyperspectral VSFG image of SDS@2β-CD. Blue and magenta colors represent areas where different spectra reside, and the corresponding spectra are plotted in (b) which are representative spectra for single pixels with signal-to-noise ratio for blue and magenta spectra of ∼56 and ∼26 respectively. The sheets in the red and blue boxes are analyzed explicitly below to extract the supramolecule tilt angles. (c) Optical image of the same area as that in (a). (d) VSFG hyperspectral image overlaid with optical image of identical area. (e) From left to right: PPS, PPP, SSP, SSS polarization resolved spectra summed over 180 and 480 pixels within the two single sheets highlighted in red rectangular boxes in (a). All spectra had a dominant feature centered at approximately 2910 cm^–1^ and a signal-to-noise ratio in the order of 1000. The spectra were fitted with multiple Voigt functions, which were represented by the shaded areas, and used for further orientation analysis.

An example of hyperspectral images of SSS polarization combination (left to right were polarization of VSFG, upconversion, and IR beams) was shown in Figure 4a. Edges of a single SDS@2β-CD sheet could be clearly seen as well as the rotational orientation of the rhombic shape inherent to the self-assembly, and the VSFG and optical images agreed well (Figure 4c,d). An atomic force microscope (AFM) was also used to image the sheets (Figure S2), and well separated single sheets are captured, with sharp edge contrast that is also seen by our VSFG microscope (Figure 4a). We noted that there was a significant improvement in the quality of VSFG images and their agreement with the optical images of the SDS@2β-CD, compared to the images in our previous publications.^31,32^ This improvement is achieved by (1) an improved sample deposition method to prepare single sheets instead of multiple sheets overlapping on top of each other and (2) the fast line-scanning VSFG microscope that allowed optimizations of image quality within a short image acquisition time and large field of view, which was not available before.

To further disentangle spectral features of the VSFG image, spectral maps were generated using the MatLab hyperspectral imaging toolbox. Two unique spectra for the SSS polarization combination were identified, highlighted in blue and magenta in Figure 4a with corresponding spectra shown in Figure 4b. Clearly, there were two types of sheets and the sheets with magenta color coding are due to sheet overlapping. In the following, we only analyze the areas highlighted with the red and blue rectangular boxes to extract tilt angles, which were single sheets identified using an optical microscope.

To extract molecular orientations, all eight lab frame VSFG polarization combinations (SSS, SSP, SPS, PSS, SPP, PSP, PPS, PPP) were collected. We summed spectra over all pixels within single sheets (pixel index shown in Figure S8), and four representative polarizations resolved VSFG spectra are shown in Figure 4e with a signal-to-noise level of 1000 (additional spectra shown in Figures S9 and S10). Each spectrum was fitted with multiple Voigt functions (shaded area, Figures 4e, S9, and S10, eq 13 in Supporting Information; detailed fitting parameters are summarized in Table S1). From the fitting, we have identified peaks at 2860 cm^–1^, 2910 cm^–1^, 2930 cm^–1^, and 2960 cm^–1^ which can be assigned to the −CH_2_ symmetric stretch, −CH_2_ asymmetric stretch, −CH_2_ Fermi resonance of p-polarized signals, and −CH_2_ Fermi resonance of s-polarized signals.^70^ In all spectra, we identified a clear peak at the 2910 cm^–1^ position, the −CH_2_ asymmetric vibration, which we used for the orientation analysis below. The signal arises only from β-CD, as when using deuterated SDS to form *d*-SDS@2β-CD complex, the signal in this region is unchanged and no signal of deuterated CH_*x*_ were observed (Figures S5 and S6).^31^ This observation is a result of the inherent chirality of β-CD which even as a dimer does not have an inversion center. Thus, the CH_*x*_ modes in β-CD are VSFG active. The CH_*x*_ modes in SDS, however, do not survive as SDS is achiral within the MSA.

### Theoretical Basis of Orientation Analysis of a Single MSA Sheet

Theoretically, VSFG spectra with different polarization combinations, which were related to the lab frame second order susceptibility, χ^(2)^_IJK_, could be expressed in terms of the molecular orientation (such as tilt angle and in-plane rotation) in the lab frame and molecular frame hyperpolarizability tensor, β_*ijk*_, through an Euler rotation (Figure S7 and eqs 5–12).^49,65,71,72^ In our measurement, the *z*-axis of the lab frame and the MSA frame were identical (i.e., sheets lying flat, but subunits may not), and the x-*y* axis of the MSA was only rotated away from their counterparts in the lab frame. Thus, in principle, we could extract the relative subunits orientation in the MSA using the lab frame VSFG spectral intensity.

**Figure 5:**
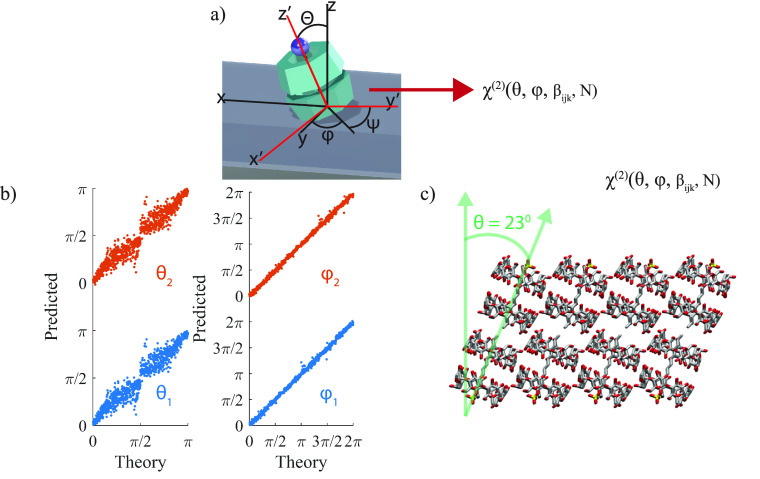
Euler transformation, neural network, and extracted tilt angles of the subunits in MSAs. (a) Euler transformation between the laboratory coordinates (*XYZ*) and the molecular coordinates (*xyz*). *z*–*y*′–*z*″ Euler rotation is performed on the molecular coordinates, with φ as the in-plane rotation angle, θ as the tilt angle, and ψ as the twist angle. (b) Neural network results for the tilt (left) and in-plane rotation (right) angles. (c) Visualization of the tilted subunits forming a sheet determined by the neural network results.

Because the SDS@2β-CD supramolecule has C_7_ symmetry, it has 13 β_*ijk*_ elements, of which only 7 are nondegenerate, *β*_*zzz*_,*β*_*xxz*_,*β*_*xzx*_,β_*zxx*_,β_*xzy*_,β_*zxy*_,β_*xyz*_ (eqs 1 and 2 in Supporting Information).^32^ We note that these hyperpolarizabilities are contributed from all CH_2_ asymmetric stretches of SDS@2β-CD supramolecule, so they together satisfy the C_7_ symmetry of the supramolecules. In this way, it is not necessary to consider hyperpolarizability of individual CH_2_ groups. Here, we also do not assume Kleinman symmetry, which has been previously reported as nonuniversal.^73,74^ Then, for the lab frame, with the NA of the condenser objective being 0.7, the axial *z*-component can be neglected,^75^ which renders 8 independent second order measurement χ^(2)^_XXX_ = χ^(2)^_SSS_, χ^(2)^_XXY_ = χ^(2)^_SSP_, χ^(2)^_XYX_ = χ^(2)^_SPS_, χ^(2)^_YXX_ = χ^(2)^_PSS_, χ^(2)^_XYY_ = χ^(2)^_SPP_, χ^(2)^_YXY_ = χ^(2)^_PSP_, χ^(2)^_YYX_ = χ^(2)^_PPS_, χ^(2)^_YYY_ = χ^(2)^_PPP_. Through an Euler rotation (Figures 5a and S7) and assuming the twist angle, ψ, is arbitrary, the lab frame χ^(2)^ could be expressed as a function of β_*ijk*_ and solid angles, resulting in a set of 8 equations (eq 5–12 in Supporting Information). One of the output equations of the Euler rotation is provided, eq 1, as an example and the rest were listed in the Supporting Information. As evident from eq 1 and Supporting Information eqs 5–12, three hyperpolarizability elements were not completely independent as they appeared as β_*xzy*_ + β_*zxy*_ and β_*xyz*_ – β_*xyz*_ grouped terms in all equations. Therefore, 7 nondegenerate hyperpolarizability elements were grouped down to 6 independent terms simplifying our set of 8 equations with 8 inputs to 8 unknowns (6 β_*ijk*_ grouped terms and two solid angles).
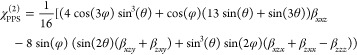
1

To extract molecular orientations in the MSA frames, we need to solve the equations based on the experimentally measured χ^(2)^ to extract θ, φ, and β_*ijk*_. To enhance the consistency of the result, we analyzed the signal on two single sheets and assumed that the supramolecular subunit in all single sheets had the same tilt angle. Two single sheets, i.e., without overlapping and with visibly different orientations, were selected as a simplified scenario (highlighted by red and blue boxes in Figures 4a and S8). The χ^(2)^ of the 2910 cm^–1^ peak of two sheets were extracted by the fitting results illustrated in Figures 4e, S10, and S11, for the orientation analysis. Based on the optical images, (Figures 4 and S8), we determined that the two single sheets are rotated approximately 60° in the *XY* plane relative to one another. Therefore, we further restrained our model with φ_2_ = φ_1_ + 60 degrees. As a result, for two sheets, we have 16 input known values (2 × 8 different polarization combinations) and 9 unknown outputs: 6 independent variables consisting of β_ijk_, the in-plane rotation φ_1_, tilt angle θ, and the relative coverage ratio *N* between sheets.

### Neural Networks To Train the Solvers and Extract Tilt Angles

This equation set was solvable; however, traditional Matlab solver either provides no solution due to less tolerance to noise, which is always present in experimental data, or runs for a long time with 2^16^ sign enumerated combinations of input and 1000 iterations for each combination.^76,77^ These limitations prohibited us from obtaining reasonable results, so we turned to a neural network approach (Figure S13).^69,78^ A training set was used to train the neural network to identify relationships between the susceptibilities and molecular details (see Method and Supporting Information). To mitigate overfitting, i.e., the model memorizes training data well but cannot generalize relationships to new data it has never encountered, we use 10% of our training set as a validation set to monitor the model loss during training (Figure S13b). It is apparent that with more training cycles the model can predict with less deviation/loss from true values, and it can also predict the validation set which contains data not used for actual training relatively well. Therefore, with experimental results that are new input for the model too, it should be able to predict with a similar error level. To quantify the error and deviation of prediction, the trained model was then tested with another data set (1000 by 27 values) generated via a similar random process (Figure S13a). Figure 5b displayed the correlation between Euler angles, i.e., molecular orientations, predicted by our model which captured the true values in the test data set well, resembling a *y* = *x* relationship with mean squared error of in-plane rotation prediction of 0.4° and that of tilt-angle at <30° region of 1.5°. Thus, our neural network based orientation solver was appropriate in predicting the molecular orientations.

Finally, we extracted molecular orientation using this method, by supplying it with the experimentally determined susceptibility values (Tables S1 and S2). Since phase information was not retrieved in our homodyne experiment, we enumerated the signs of all 16 susceptibility values when supplying them to the model and selected the predicted results with the smallest mean squared error of susceptibilities. By calculating the mean squared errors of the predicted normalized susceptibility values and the experimentally determined ones, the smallest mean squared error we can obtain is 0.02, whose corresponding predicted tilt angle away from the lab frame *z*-axis is 23 ± 1.5° (Supporting Information Table S2 and Figure 5c). To the best of our knowledge, this is the first work using neural network to relate VSFG spectral observables back to physical properties of molecules, while other studies involving machine learning in the SFG field mainly focus on assisting peak fitting/assignment and sole spectral analysis.^79−81^

From the polarization-dependent equations we could see that if the tilt angle was 0°, all susceptibility terms on the left side of the equations would be zero, which did not agree with the strong SFG signal, implying nonzero susceptibilities of the SDS@2β-CD system. On the other hand, using the literature reported in-plane unit cell parameters of the SDS@2β-CD system,^11^ we could visually demonstrate that when the tilt angle was 30°, the space between subunits was tight, and when the tilt angle was 45–60°, SDS@2β-CD subunits would collide with each other (Figure S12). Hence, The results retrieved from the neural network mode, i.e., the SDS@2β-CD subunits were tilted slightly at 23 ± 1.5°, was appropriate and consistent with existing structural knowledge of the self-assembly. The tilt angle result is further validated when compared with results from X-ray diffraction. The XRD results indicate the stacking height of 4 SDS@2β-CD (a unit cell) to be 2.9 nm, while if the 4 SDS@2β-CDs sit straight up, their height should be 3.1 nm. To make the height to be 2.9 nm, an angle of approximately 20° is necessary (further explained in Supporting Information section I and Figure S4). Moreover, the AFM height profile shows a gradient height change across the edge and the edge incline/tilt toward sheet center, which could also be due to the stacking of tilted subunits. Comparable to the well-studied self-assembled monolayer on metal substrates, we believed the driving force of the tilting could be the interplay of intermolecular interactions (such as hydrogen bonds) among subunits and binding behaviors between subunits and substrate.^22,27,82,83^

It had been widely studied that the tilt of molecules within monolayers commonly existed in self-assembled materials and potentially adjusted the conduction,^84,85^ wetting,^86,87^ or mechanical properties.^84,88,89^ With SDS@2β-CD as an important biomimetic motif, the tilt angle resolved in our neural network approach sheds light on how the subunits pack within the self-assembly and provides a protocol to study other MSA systems structure–properties correlations. For example, when the supramolecule units tilt, the top part of one unit overlaps with the bottom part of another unit, creating a fish scale type of structure, increasing the mechanical stability, compared to if the unit sits straight up. Moreover, as the tilting is influenced by the interaction of the subunits and hydration level, future works on humidity dependent packing of the system might unravel how the chemical environments affect the self-assembly process and could be very interesting and significant for the drug delivery field^90^ as the release of target molecules highly relies on the biological environment.

It is noteworthy that such information is difficult to obtain with IR microscope. Based on the C7 symmetry of the SDS@2β-CD supramolecule, only μ_*z*_ is nonvanishing in molecular frame. The dipole moment in the lab frame can be expressed as a vector (cos ϕ sin θμ_*z*_ sin ϕ sin θμ_*z*_ cos θμ_*z*_) via the aforementioned Euler transformation. To extract tilt angle θ, we have to measure the *Z* component cos θμ_*z*_ in the lab frame, which is practically difficult as it requires the incident IR beam to have a large polarization component along the surface normal. Furthermore, with 3 unknowns (φ, Θ, μ_*z*_) and only 2 knowns (P and S polarizations of IR beam), it is an underdetermined problem, which still requires the multisheet approach demonstrated in our neural network approach.

## Conclusion

We note that there are a couple of limitations of the current microscope. First. The spatial resolution is only 1.6 μm, which limits its utility to imaging smaller domains. This limitation could be improved by using higher NA objective lens.^50^ Second, the current sensitivity might not be able to image a self-assembled monolayer. This limitation is partly because the electromagnetic field projection on the *z* axis is too small, which always contributes strongly to the self-assembled monolayers. Thus, it is possible to image monolayers by tilting the sample relative to the beam propagation directions. However, it will detoriate the spatial resolution. Nevertheless, should these limitations be overcome, the present fast line-scanning and neural network analysis method can be readily applied. Indeed, the neural network analysis can be applied to any typical SFG spectroscopy study, when the orientation analysis becomes difficult.

The molecular self-assembly formed from a mixture of β-CD and SDS in water was analyzed using a line-scanning hyperspectral VSFG microscope and neural network. A 1D resonant scanner coupled to a CCD/spectrograph increases image collection speed 10-fold with simultaneous spectral information. This development enables polarization resolved VSFG images of single MSA sheets, which were analyzed by a neural network approach. The analysis revealed that the supramolecular subunits are tilted at around 23 ± 1.5° in the SDS@2β-CD MSA frame. Such information could help us further understand the structure and intermolecular interactions of other biomimetic morphologies that the subunits construct. This information became available because of the power of VSFG microscopy to extract spatially resolved, spectral information of the sheets.

## Supporting Information Available

Euler rotation and spectra fitting equations, XRD data, neural network details, spectra analysis with MATLAB hyperspectral imaging toolbox, and fitting results (PDF)
