# Supplementary material for: Imaging Orientation of a Single Molecular Hierarchical Self-Assembled Sheet: The Combined Power of a Vibrational Sum Frequency Generation Microscopy and Neural Network
Source: J Phys Chem B. 2022 Sep 13;126(37):7192–201. doi: 10.1021/acs.jpcb.2c05876 (PMC9511492; doi:10.1021/acs.jpcb.2c05876)
Supplement: Supplementary file 1 — jp2c05876_si_001.pdf [file jp2c05876_si_001.pdf]

Imaging Orientation of a Single Molecular Hierarchical Self-Assembled Sheet: The Combined Power of a Vibrational Sum Frequency Generation Microscopy and Neural Network

*Jackson C. Wagner[#,1], Zishan Wu[#,1], Haoyuan Wang[1,≠], and Wei Xiong[1,2,3]**

[1]Department of Chemistry and Biochemistry, UC San Diego, La Jolla, CA, 92093, U.S.A.

[2]Materials Science and Engineering Program, UC San Diego, La Jolla, CA, 92093, U.S.A.

[3]Department of Electrical and Computer Engineering, UC San Diego, La Jolla, CA 92093, U.S.A.

Email: w2xiong@ucsd.edu

[#] These authors contributed equally to this work.

[≠] *Current address: Intel Corporation, Rio Rancho, NM 87124, U.S.A.*

I. Sample Preparation and Characterizations

All chemicals were purchased from Sigma Aldrich without further purification. Well-separated SDS@2β-CD sheets are prepared by spin-coating 5μL of the SDS@2β-CD water suspension at 10,000 rpm spin rate onto glass coverslips (1 in by 1 in, 170 ± μm thickness). The spin-coating parameters were optimized to minimize sheets overlapping, observable under optical microscope (Fig. S1). Atomic force microscope (AFM) was used to image the sheets and obtain their height profiles. Height is consistent within each sheet and is measured to be 110 nm on average (Fig.S2-3). From AFM, we can also observe tilted edges which could be related to the supramolecular tilt angles reported here (Fig.S3).

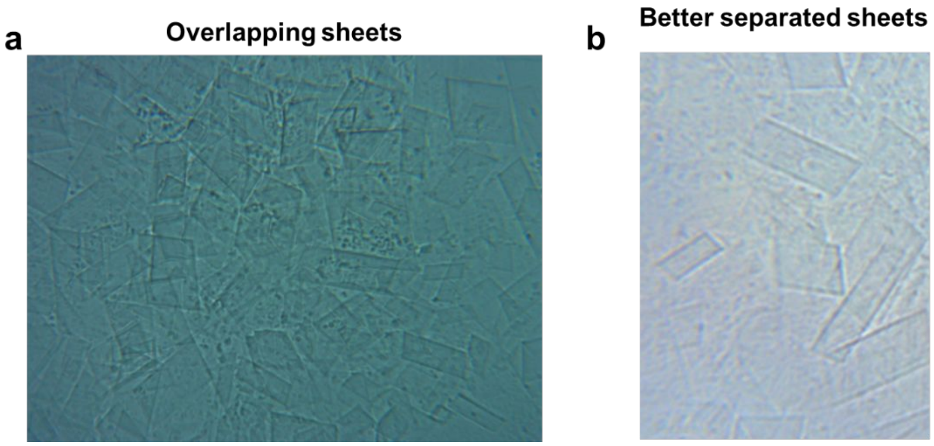

Figure S1 *Comparison of sparseness of SDS@2β-CD sheets with different spincoating parameters.* a) Obvious sheet overlapping when spincoating 40 μL of suspension at 5,000 rpm spin speed. b) Less sheet overlapping and sparser sheets when spincoating 5 μL of suspension at 10,000 rpm spin speed. Optical images are taken with 40X objective.

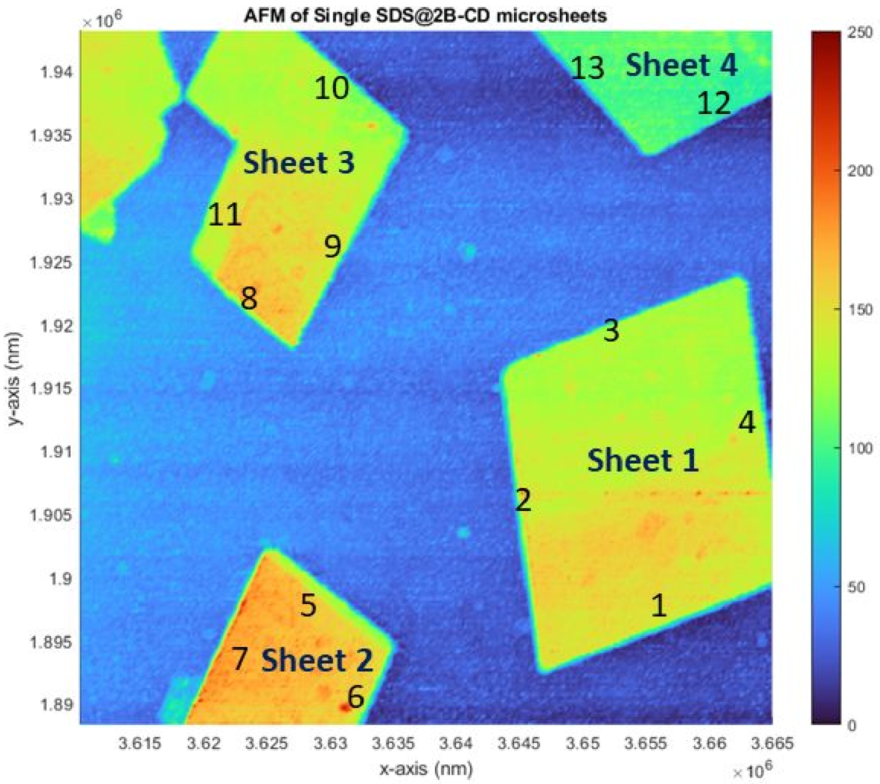

Figure S2 *AFM image of well-separated sheets. X and Y axis are in 10^6 nm scale and intensity axis is in nm unit. 4 Sheets are labeled as well as edge faces. Height profile for each sheet is shown in Figure S3.*

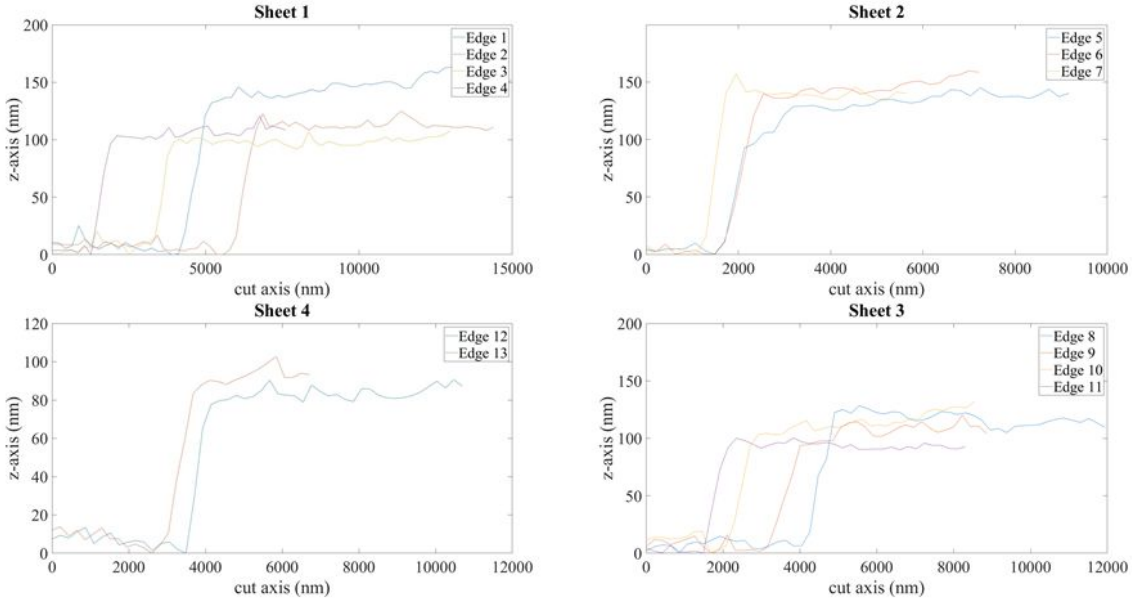

Figure S3 *Height profile for all edges of all sheets labeled in Figure S2. Average height for all sheets is approximately 110nm.*

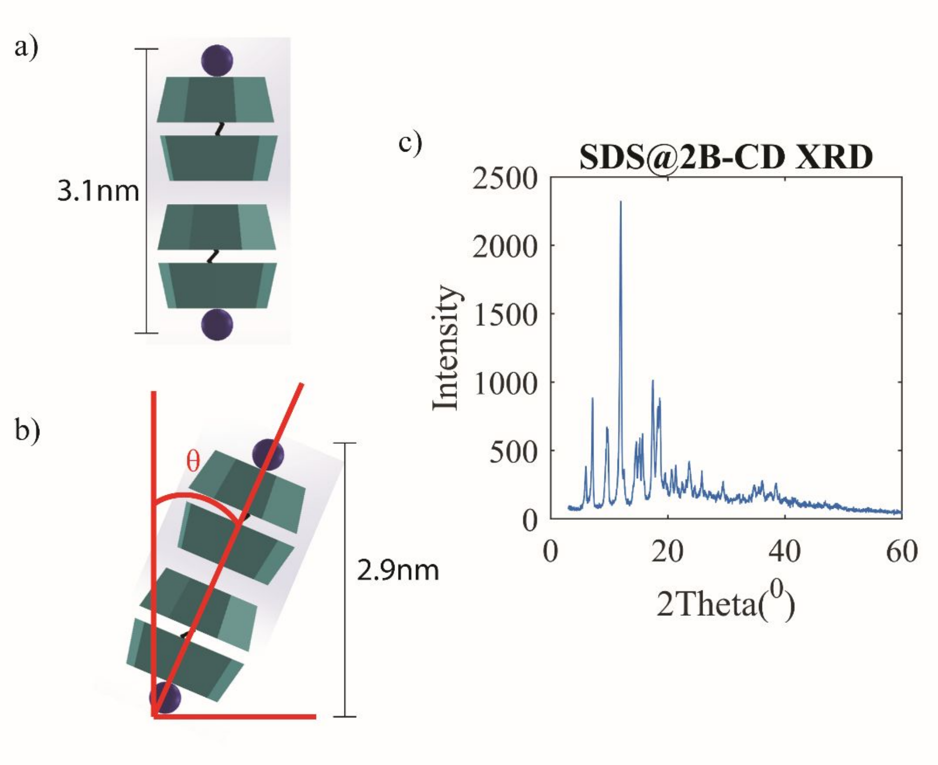

Figure S4 **a)** height of a single SDS @2β-CD subunit with straight up stacking, reported by the Jiang group.[1] **b)** tilted subunit according to calculations below based on XRD data **c)** XRD of SDS @2β-CD sheets collected via centrifuge. Lowest 2theta angle corresponds to 6.02^0.

X-ray Diffraction (XRD) was used to obtain the structural details of the sheets collected via centrifuge. Using the lowest 6.02° 2theta angle obtained via XRD, shown in Figure S4c, we can calculate the lattice parameters of the SDS @2β-CD assembly using the following Bragg's equation:

$$d_{hkl} = \frac{\lambda}{2sin\theta}$$

Where,

$$\lambda = 0.15406\ nm \text{ (Cu } k_\alpha \text{ X - Ray source frequency)}$$

$$\theta = 6.02°$$

Which gives us d spacing of 0.73 nm. Since there are 4 β-CD per subunit (Fig. S4a-b), and assuming the lattice parameter are the distance from the two adjacent β-CD units, we multiply by 4 to get 2.9nm as the height of one subunit. It was reported that the height of two SDS@2β-CD along the long axis is 3.1 nm.[1] Therefore, as labeled in Figure S4b, it is likely that subunits are tilted relative to the surface normal, which can be calculated to be 21° of a tilt angle through trigonometry (*i.e.*: $\theta = \cos^{-1}\frac{2.9}{3.1}$) depicted in Figure S4b.

To determine chemically profile the origins of VSFG signals broadband linear, FTIR, and nonlinear spectra, VSFG, were obtained for the MSA with and without deuterated SDS. Spectra for FTIR and broadband VSFG are shown in Figs S5 and S6. For FTIR, in the 2800cm^{-1} to 3000cm^{-}

^{1} region, MSA with undeuterated SDS shows extra peak, likely originated from SDS alkyl chain C-H bonding, which is absent in the one with deuterated SDS since all C-H is replaced by C-D. A more significant difference is the appearance of the -CD feature in the ~2000cm^{-1}-2200cm^{-1} region, which is contributed by the deuterated SDS alkyl chain. It is reasonable to observe SDS contributions in FTIR, which is a bulk sensitive technique.

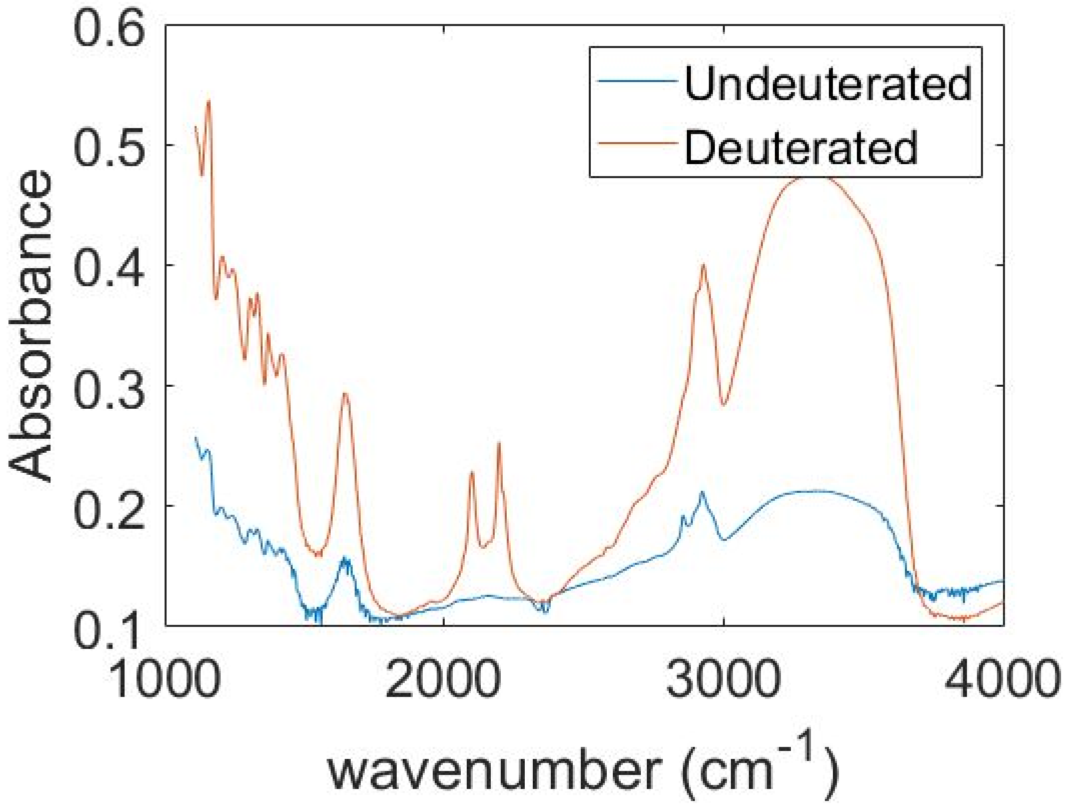

Figure S5 FTIR overlay of the MSA with undeuterated and deuterated SDS. No major qualitative differences are observed between the two sample conditions besides the appearance of the -CD feature in the deuterated SDS MSA spectrum in the 2000cm^{-1}-2200cm^{-1} region corresponding to the $-CD_x$ in d-SDS.

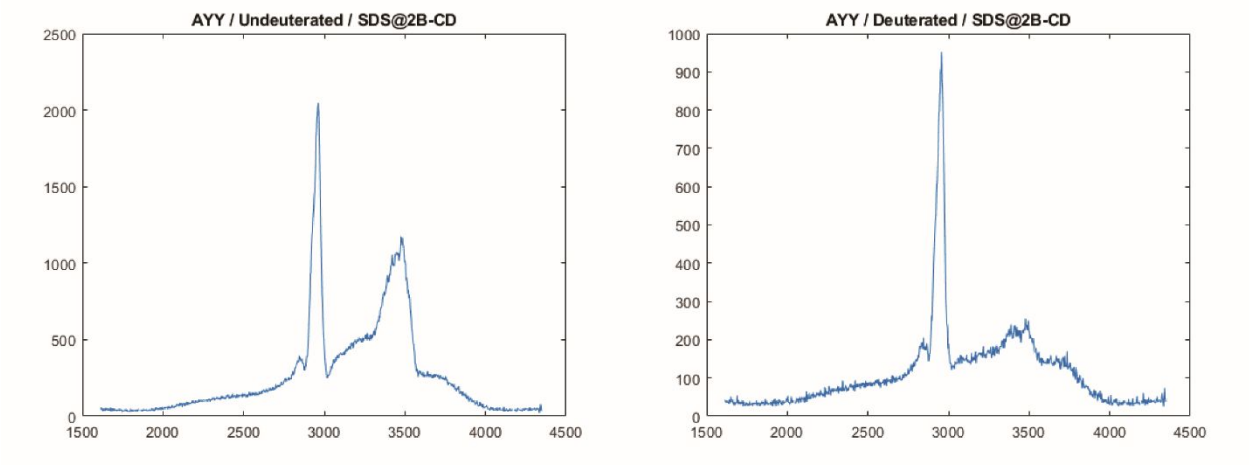

Figure S6 Broadband VSFG spectra for deuterated and undeuterated MSA samples. Comparing the deuterated FTIR spectrum with the deuterated VSFG spectrum we observe no appearance of the -CD_x feature found in the 2000cm^{-1}-2200cm^{-1} region of the FTIR. This confirms that SDS does not contribute to the VSFG spectra and only chiral β-CD would contribute.

On the contrary, in the broadband VSFG analysis, there is no obvious contribution from SDS to the overall VSFG signal, with no apparent C-D feature in the ~2000cm^{-1}-2200cm^{-1} region, and no difference in the 2800cm^{-1} to 3000cm^{-1} region. Thus, the VSFG signal mainly arises from the chirality of β-CD, and even with the dimer configuration the chiral nature is still preserved. Yet SDS signal is likely canceled out due to inversion symmetry in dimer configuration.

II. Euler Rotation

The following matrix represents the 13 surviving terms of the hyperpolarizability tensor β_{ijk} in a C7 point group. The first matrix on the left depicts terms $\beta_{i,j,x}$, the second $\beta_{i,j,y}$ and the third $\beta_{i,j,z}$.

$$\begin{bmatrix} 0 & 0 & \beta_{xzx} \\ 0 & 0 & \beta_{yzx} \\ \beta_{zxx} & \beta_{zyx} & 0 \end{bmatrix} \begin{bmatrix} 0 & 0 & \beta_{xzy} \\ 0 & 0 & \beta_{yzy} \\ \beta_{zxy} & \beta_{zyy} & 0 \end{bmatrix} \begin{bmatrix} \beta_{xxz} & \beta_{xyz} & 0 \\ \beta_{yxz} & \beta_{yyz} & 0 \\ 0 & 0 & \beta_{zzz} \end{bmatrix} \qquad \textbf{Eq. 1}$$

Taking into account degenerate terms, $\beta_{xxz} = \beta_{yyz}$, $\beta_{xzx} = \beta_{yzy}$,$\beta_{zxx} = \beta_{zyy}$, $\beta_{xzy} = -\beta_{yzx}$,$\beta_{zxy} = -\beta_{zyx}$, $\beta_{xyz} = -\beta_{yxz}$. The hyperpolarizability tensor for the MSA reduces to 7 unknown hyperpolarizability elements.

$$\begin{bmatrix} 0 & 0 & \beta_{xzx} \\ 0 & 0 & -\beta_{xzy} \\ \beta_{zxx} & -\beta_{zxy} & 0 \end{bmatrix} \begin{bmatrix} 0 & 0 & \beta_{xzy} \\ 0 & 0 & \beta_{xzx} \\ \beta_{zxy} & \beta_{zxx} & 0 \end{bmatrix} \begin{bmatrix} \beta_{xxz} & \beta_{xyz} & 0 \\ -\beta_{xyz} & \beta_{xxz} & 0 \\ 0 & 0 & \beta_{zzz} \end{bmatrix} \qquad \textbf{Eq. 2}$$

The correlations of 2nd-order susceptibility tensor $\chi_{ijk}^{(2)}$ extracted from lab-frame measurement and 2nd-order hyperpolarizability tensor β_{ijk} in the molecular frame can be expressed by an Euler rotation (Fig. S7).

$$R = \begin{bmatrix} \cos\varphi\cos\theta\cos\psi - \sin\varphi\sin\psi & -\cos\psi\sin\varphi - \cos\varphi\cos\theta\sin\psi & \cos\varphi\sin\theta \\ \cos\varphi\sin\psi + \cos\theta\cos\psi\sin\varphi & \cos\varphi\cos\psi - \cos\theta\sin\varphi\sin\psi & \sin\varphi\sin\theta \\ -\cos\psi\sin\theta & \sin\theta\sin\psi & \cos\theta \end{bmatrix}$$

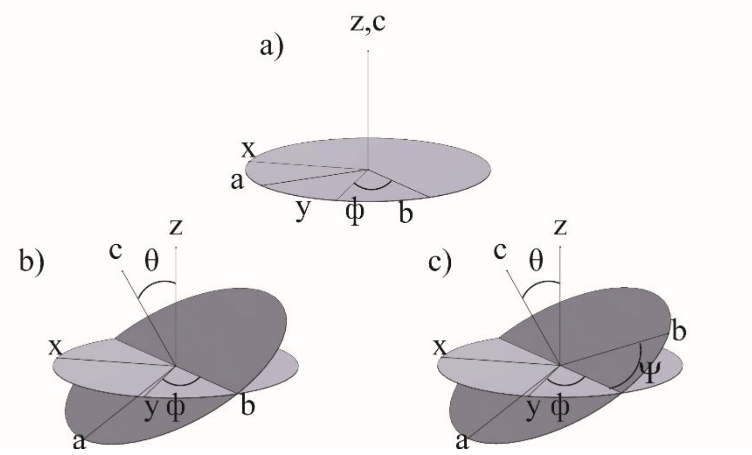

Figure S7 Visual representation of the Euler rotation transform. a) the object (self-assembly) is rotated about the optical axis Z of the set-up by an angle φ (in-plane rotation) b) the same object is then tilted about the new b axis by θ c) the sample is again rotated about the new molecular c-axis by ψ. which is integrated over.

And following the transform process,

$$\begin{bmatrix} x \\ y \\ z \end{bmatrix} = R * \begin{bmatrix} x' \\ y' \\ z' \end{bmatrix} = \begin{bmatrix} R_{xx'} & R_{xy'} & R_{xz'} \\ R_{yx'} & R_{yy'} & R_{yz'} \\ R_{zx'} & R_{zy'} & R_{zz'} \end{bmatrix} \begin{bmatrix} x' \\ y' \\ z' \end{bmatrix} \qquad \textbf{Eq. 4}$$

The output from the arbitrary Euler rotation of the molecular frame hyperpolarizability tensor to the macroscopic lab frame second order nonlinear susceptibility is detailed in **Eq. 5-12**. With our incident laser beam focused via a reflective objective with NA <0.8, we can neglect the z-component and have the S and P polarizations correspond to X and Y directions in the sample plane, *i.e.* $\chi_{YYY}^{(2)} = \chi_{PPP}^{(2)}$.

$$\chi_{YYY}^{(2)} = \chi_{PPP}^{(2)} = \sin(\theta)\sin(\varphi)\left(\cos^2(\varphi) + \cos^2(\theta)\sin^2(\varphi)\right)\left(\beta_{xxz} + \beta_{xzx} + \beta_{zxx}\right) + \sin^3(\theta)\sin^3(\qquad \textbf{Eq. 5}$$

$$\chi_{YYX}^{(2)} = \chi_{PPS}^{(2)} = \frac{1}{16}[(4\cos(3\varphi)\sin^3(\theta) + \cos(\varphi)(13\sin(\theta) + \sin(3\theta))\beta_{xxz} - 8\sin \left(\sin(2\theta)(\beta_{xzy} + \beta_{zxy}) + \sin^3(\theta)\sin(2\varphi)(\beta_{xzx} + \beta_{zxx} - \beta_{zzz})\right)] \qquad \textbf{Eq. 6}$$

$$\chi_{YXX}^{(2)} = \chi_{PSS}^{(2)} = \frac{1}{8}(-4\cos(\varphi)\sin(2\theta)(\beta_{xyz} + \beta_{xzy}) + \sin(\theta)\sin(\varphi) \left((7 + \cos(2\theta))\beta_{zxx} - 2\sin^2(\theta)(\beta_{xxz} + \beta_{xzx} - \beta_{zxx})\right) - 2\sin^3(\theta)\sin(3\varphi)(\beta_{xxz} - {-}\beta_{zzz})) \qquad \textbf{Eq. 7}$$

$\chi^{(2)}_{YXY} = \chi^{(2)}_{PSP} = \frac{1}{16}(- 8sin^3(\theta)sin\,(\varphi)sin\,(2\varphi)\beta_{xxz} + (4cos\,(3\varphi)sin^3(\theta) + cos\,(\varphi)($ $+ 8sin\,(\varphi)(sin\,(2\theta)(-\beta_{xyz} + \beta_{zxy}) + sin^3(\theta)sin\,(2\varphi)(-\beta_{zxx} + \beta_{zzz})))$	**Eq. 8**
$\chi^{(2)}_{XXX} = \chi^{(2)}_{SSS} = cos\,(\varphi)sin\,(\theta)((cos^2(\theta)cos^2(\varphi) + sin^2(\varphi))(\beta_{xxz} + \beta_{xzx} + \beta_{zxx}) + cos^2(\varphi)sin^{[illegible]}$	**Eq. 9**
$\chi^{(2)}_{XXY} = \chi^{(2)}_{SSP} = \frac{1}{8}[sin\,(\theta)((7 + cos\,(2\theta))sin\,(\varphi) - 2sin^2(\theta)sin\,(3\varphi))\beta_{xxz} + 4cos\,(\varphi)(sin\,(2\theta)$ $- sin^3(\theta)sin\,(2\varphi)(\beta_{xzx} + \beta_{zxx} - \beta_{zzz}))]$	**Eq. 10**
$\chi^{(2)}_{XYY} = \chi^{(2)}_{SPP} = sin\,(\theta)(- cos\,(\varphi)sin^2(\theta)sin^2(\varphi)\beta_{xxz} - cos\,(\varphi)sin^2(\theta)sin^2(\varphi)\beta_{xzx} + cos\,(\theta)s[illegible]$ $+ \beta_{xzy}) + \frac{1}{8}((7 + cos\,(2\theta))cos\,(\varphi) + 2cos\,(3\varphi)sin^2(\theta))\beta_{zxx} + cos\,(\varphi)sin^2(\theta)[illegible]$	**Eq. 11**
$\chi^{(2)}_{XYX} = \chi^{(2)}_{SPS} = \frac{1}{8}(- 8cos^2(\varphi)sin^3(\theta)sin\,(\varphi)\beta_{xxz} + sin\,(\theta)((7 + cos\,(2\theta))sin\,(\varphi) -$ $cos\,(\varphi)(sin\,(2\theta)(\beta_{xyz} - \beta_{zxy}) + sin^3(\theta)sin\,(2\varphi)(- \beta_{zxx} + \beta_{zzz})))$	**Eq. 12**

III. Spectral fitting

Spectra for sheet 1 and sheet 2 were obtained for each polarization by summing spectra over all pixels within the regions highlighted in **Fig. S8**.

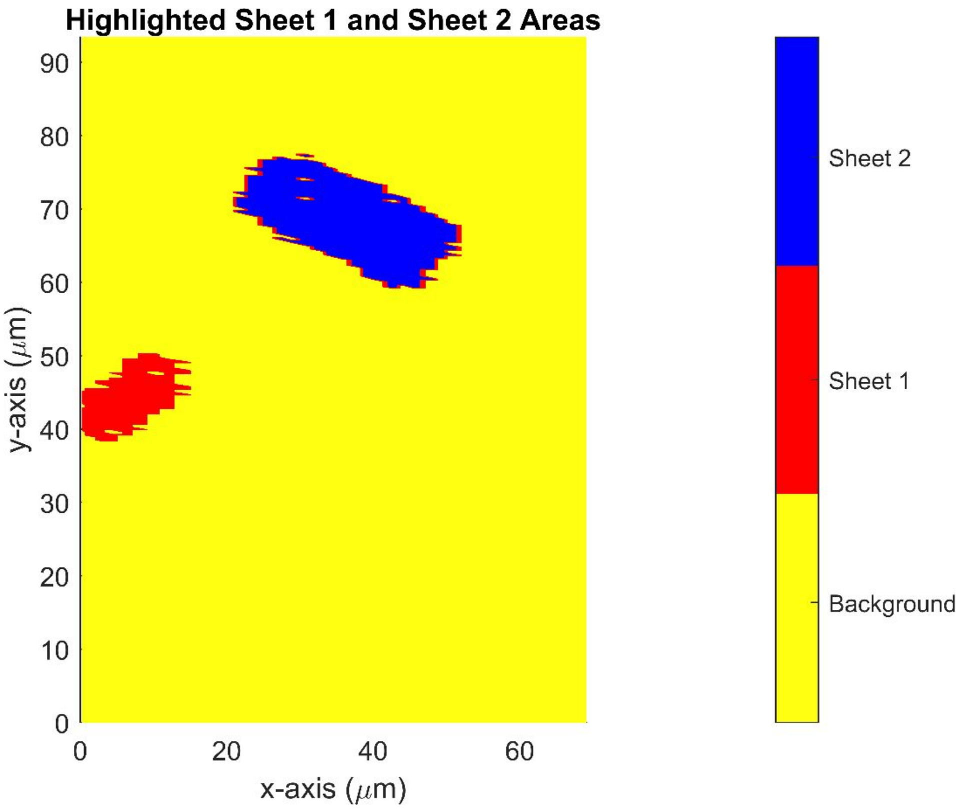

Figure S8 Highlighted regions for sheet 1 and sheet 2 where spectra of all pixels within the sheets are summed to obtain integrated spectra shown in Fig. S9-10 as well as Fig. 4e in main text.

Spectra with high signal to noise ratio (SNR) were collected for all 8 polarization combinations and fit to a Voigt profile, shown in Eq. 13 which is a convolution of a Gaussian lineshape that accounts for inhomogenous broadening, and a Lorentzian lineshape that accounts for

homogeneous broadening, with $\chi^{(2)}_{NR}$, ψ, A_υ, ψ_υ, ω_{IR}, ω_L, $\Gamma_{L,\upsilon}$ and, Γ_υ as the second order nonlinear susceptibility of the nonresonant interaction, the phase of the nonresonant signal, oscillator strength, phase of the resonant signal, IR spectral coverage, oscillator center frequency, Lorentzian linewidth, and Gaussian linewidth respectively.

$$I(\omega_{IR}) \propto \left| \chi_{NR}^{(2)} e^{i\psi} + \sum_v \int \frac{A_v e^{i\psi_v}}{\omega_{IR} - \omega_L + i\Gamma_{L,v}} e^{-\left(\frac{\omega_L - \omega_v}{\Gamma_v}\right)^2} d\omega_L \right|^2 \qquad \textbf{Eq. 13}$$

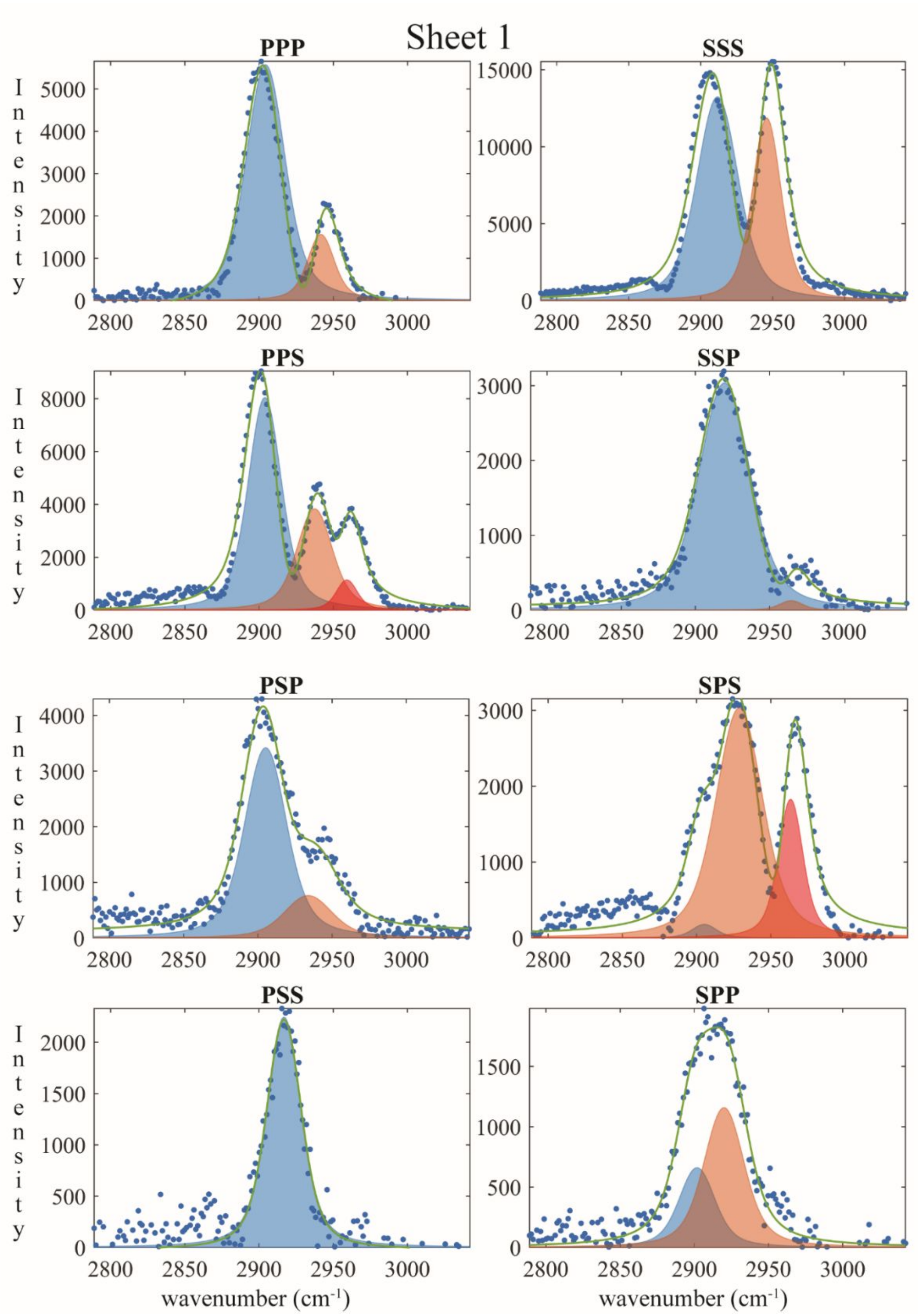

Figure S9 Fitted spectra for sheet 1 generated from the integration of the red region in Fig S8. Fitted parameters are shown in Table S1 and are used for further orientation analysis with neural network

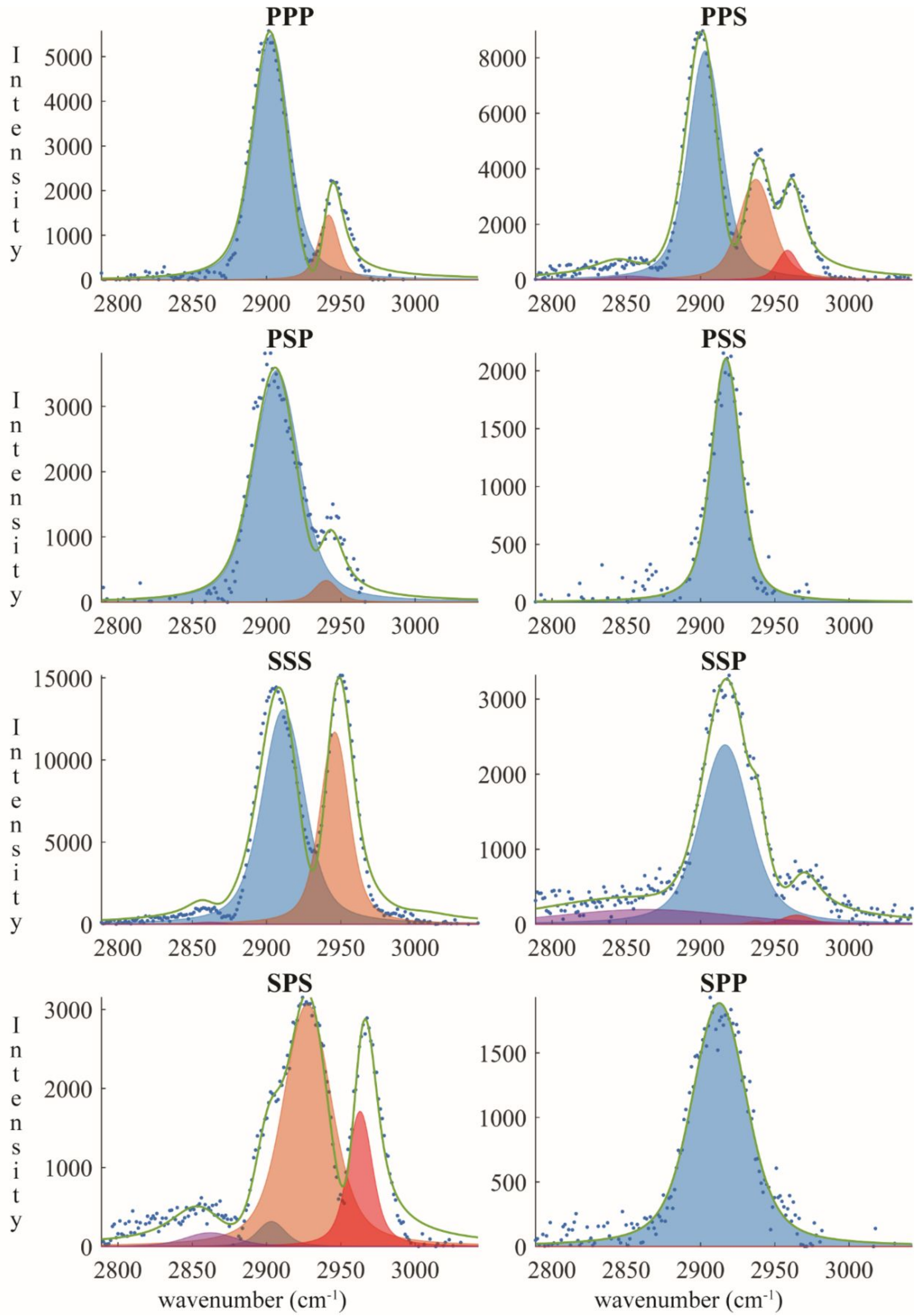

SI Figure S10 Fitted spectra for sheet 2 generated from the integration of the blue region in Fig S8. *Fitted parameters are shown in Table S1 and are used for further orientation analysis with neural network.*

Table S1 Voigt Fitting results performed in MatLab for both Sheet 1 and Sheet 2 highlighted in Fig. S8

Sheet 1

PPP				
	Lower Boundary	Initial Conditions	Upper Boundary	Equilibrium
Noise	0	0.0001	2000	2.35E-14
Non-Resonant Background	0	50	2000	1.40E+00
A_1	0	1000	2000	648.6126
ω_1	2885	2901	2915	2903.19
Γ_1	2	2	2	2
$\Gamma_{1,1}$	0	10	50	13.1811
A_2	0	500	20000	201.6033
ω_2	2930	2944	2960	2941.787
Γ_2	2	2	2	2
$\Gamma_{1,2}$	0	10	50	6.966383

PPS				
	Lower Boundary	Initial Conditions	Upper Boundary	Equilibrium
Noise	0	0.0001	2000	2.26E-14
Non-Resonant Background	0	50	2000	2.26E-14
A_1	0	5440	5440	132.2091
ω_1	2840	2856	2870	2852.361
Γ_1	2	2	2	2
$\Gamma_{1,1}$	0	30	50	17.22956
A_2	0	1000	2000	759.3557
ω_2	2885	2897	2915	2903.107
Γ_2	2	2	2	2
$\Gamma_{1,2}$	0	10	50	12.47629
A_3	0	500	20000	502.2201
ω_3	2925	2938	2955	2937.178
Γ_3	2	2	2	2
$\Gamma_{1,3}$	0	10	50	12.44146
A_4	0	432	20000	173.6801
ω_4	2945	2958	2975	2958.511
Γ_4	2	2	2	2
$\Gamma_{1,4}$	0	10	100	7.00479

PSP				
	Lower Boundary	Initial Conditions	Upper Boundary	Equilibrium
Noise	0	0.0001	2000	4.43E-14
Non-Resonant Background	0	50	2000	0.60581
A_1	0	1000	2000	654.9771
ω_1	2885	2901	2915	2906.835
Γ_1	2	2	2	2
$\Gamma_{1,1}$	0	10	50	17.16326
A_2	0	500	20000	110.9372
ω_2	2940	2947	2955	2940
Γ_2	2	2	2	2
$\Gamma_{1,2}$	0	10	50	0

PSS				
	Lower Boundary	Initial Conditions	Upper Boundary	Equilibrium
Noise	0	0.0001	2000	3.45E-14
Non-Resonant Background	0	50	2000	3.91E-09
A_1	0	500	20000	337.3558
ω_1	2905	2920	2935	2917.106
Γ_1	2	2	2	2
$\Gamma_{1,1}$	0	10	50	10.67493

SSS				
	Lower Boundary	Initial Conditions	Upper Boundary	Equilibrium
Noise	0	0.0001	2000	1.01E-13
Non-Resonant Background	0	50	2000	9.75E-07
A_1	0	5440	5440	78.92454
ω_1	2845	2858	2875	2861.585
Γ_1	2	2	2	2
$\Gamma_{1,1}$	0	30	50	9.537006
A_2	0	1000	2000	1175.497
ω_2	2890	2905	2920	2911.369
Γ_2	2	2	2	2
$\Gamma_{1,2}$	0	10	50	15.89421

A_3	0	500	20000	790.198
ω_3	2935	2950	2965	2945.876
Γ_3	2	2	2	2
$\Gamma_{1,3}$	0	10	50	10.58672
A_4	0	432	20000	43.99274
ω_4	2970	2986	3000	3000
Γ_4	2	2	2	2
$\Gamma_{1,4}$	0	10	50	15.76194

SSP

	Lower Boundary	Initial Conditions	Upper Boundary	Equilibrium
Noise	0	0.0001	2000	41.8
Non-Resonant Background	0	50	2000	0.0
A_1	0	0.0001	2000	5.14E-14
ω_1	0	50	2000	2.42E-14
Γ_1	0	5440	5440	558.2505
$\Gamma_{1,1}$	2855	2870	2885	2863.177
A_2	2	2	2	2
ω_2	0	30	200	68.02372
Γ_2	0	1000	2000	580.6063
$\Gamma_{1,2}$	2900	2915	2930	2916.514
A_3	2	2	2	2
ω_3	0	10	110	18.7311
Γ_3	0	500	20000	26.31386
$\Gamma_{1,3}$	2925	2940	2955	2937.074
A_4	2	2	2	2
ω_4	0	10	50	4.327213
Γ_4	0	432	20000	81.39117
$\Gamma_{1,4}$	2960	2975	2990	2964.828

SPS

	Lower Boundary	Initial Conditions	Upper Boundary	Equilibrium
Noise	0	0.0001	2000	2.34E-14
Non-Resonant Background	0	50	2000	2.34E-14
A_1	0	5440	5440	160.6936
ω_1	2845	2863	2875	2862.17
Γ_1	2	2	2	2

$\Gamma_{1,1}$	0	30	50	19.20785
A_2	0	1000	2000	124.0668
ω_2	2885	2901	2915	2903.32
Γ_2	2	2	2	2
$\Gamma_{1,2}$	0	10	50	9.988176
A_3	0	500	20000	640.0068
ω_3	2910	2924	2940	2927.49
Γ_3	2	2	2	2
$\Gamma_{1,3}$	0	10	50	18.15893
A_4	0	432	20000	261.0439
ω_4	2950	2967	2980	2962.946
Γ_4	2	2	2	2
$\Gamma_{1,4}$	0	10	50	8.805774

SPP				
	Lower Boundary	Initial Conditions	Upper Boundary	Equilibrium
Noise	0	0.0001	2000	2.37E-14
Non-Resonant Background	0	50	2000	3.97E-14
A_1	0	1000	2000	566.5574
ω_1	2900	2914	2930	2912.596
Γ_1	2	2	2	2
$\Gamma_{1,1}$	0	10	50	20.80821
A_2	0	500	20000	5.62E-11
ω_2	2950	2962	2980	2980
Γ_2	2	2	2	2
$\Gamma_{1,2}$	0	10	50	50

Sheet 2

PPP				
	Lower Boundary	Initial Conditions	Upper Boundary	Equilibrium
Noise	0	0.0001	2000	2.37E-14
Non-Resonant Background	0	50	2000	5.56E-02
A_1	0	1000	2000	426.5297
ω_1	2885	2901	2915	2903.179
Γ_1	2	2	2	2
$\Gamma_{1,1}$	0	10	50	10.74409

A_2	0	500	20000	257.9949
ω_2	2930	2944	2960	2934.513
Γ_2	2	2	2	2
$\Gamma_{1,2}$	0	10	50	13.20489

PPS

	Lower Boundary	Initial Conditions	Upper Boundary	Equilibrium
Noise	0	0.0001	2000	7.10E-13
Non-Resonant Background	0	50	2000	4.62E-07
A_1	0	5440	5440	109.3865
ω_1	2840	2856	2870	2840.632
Γ_1	2	2	2	2
$\Gamma_{1,1}$	0	30	50	21.62556
A_2	0	1000	2000	989.0181
ω_2	2885	2897	2915	2901.614
Γ_2	2	2	2	2
$\Gamma_{1,2}$	0	10	50	11.31966
A_3	0	500	20000	809.3231
ω_3	2925	2938	2955	2937.471
Γ_3	2	2	2	2
$\Gamma_{1,3}$	0	10	50	9.36711
A_4	0	432	20000	256.1476
ω_4	2945	2958	2975	2956.021
Γ_4	2	2	2	2
$\Gamma_{1,4}$	0	10	100	7.116477

PSP

	Lower Boundary	Initial Conditions	Upper Boundary	Equilibrium
Noise	0	0.0001	2000	2.34E-14
Non-Resonant Background	0	50	2000	7.62E-11
A_1	0	1000	2000	746.4166
ω_1	2885	2901	2915	2909.247
Γ_1	2	2	2	2
$\Gamma_{1,1}$	0	10	50	15.70295
A_2	0	500	20000	3.65E-13
ω_2	2940	2947	2955	2955
Γ_2	2	2	2	2

$\Gamma_{1,2}$	0	10	50	0.038674

PSS

	Lower Boundary	Initial Conditions	Upper Boundary	Equilibrium
Noise	0	0.0001	2000	4.32E-09
Non-Resonant Background	0	50	2000	5.23E-10
A_1	0	500	20000	273.3897
ω_1	2940	2952	2970	2951.743
Γ_1	2	2	2	2
$\Gamma_{1,1}$	0	10	50	7.351582

SSS

	Lower Boundary	Initial Conditions	Upper Boundary	Equilibrium
Noise	0	0.0001	2000	4.32E-14
Non-Resonant Background	0	50	2000	4.62E-12
A_1	0	5440	5440	225.75
ω_1	2840	2856	2870	2840
Γ_1	2	2	2	2
$\Gamma_{1,1}$	0	30	50	22.6209
A_2	0	1000	20000	2362.029
ω_2	2885	2907	2915	2912.566
Γ_2	2	2	2	2
$\Gamma_{1,2}$	0	10	50	17.50038
A_3	0	500	20000	1254.826
ω_3	2930	2948	2960	2944.168
Γ_3	2	2	2	2
$\Gamma_{1,3}$	0	10	50	9.792042
A_4	0	432	20000	111.8797
ω_4	2970	2984	3000	3000
Γ_4	2	2	2	2
$\Gamma_{1,4}$	0	10	100	18.19438

SSP

	Lower Boundary	Initial Conditions	Upper Boundary	Equilibrium

Noise	0	0.0001	2000	3.90E-14
Non-Resonant Background	0	50	2000	4.34E-12
A_1	0	1000	2000	738.4495
ω_1	2880	2897	2910	2902.692
Γ_1	2	2	2	2
$\Gamma_{1,1}$	0	10	110	12.16319
A_2	0	500	20000	522.6157
ω_2	2925	2940	2955	2936.127
Γ_2	2	2	2	2
$\Gamma_{1,2}$	0	10	50	9.869057
A_3	0	432	20000	142.0535
ω_3	2950	2962	2980	2957.366
Γ_3	2	2	2	2
$\Gamma_{1,3}$	0	10	20	6.05511

SPS				
	Lower Boundary	Initial Conditions	Upper Boundary	Equilibrium
Noise	0	0.0001	2000	2.37E-14
Non Resonant Background	0	50	2000	2.61E-14
A_1	0	5440	5440	70.67376
ω_1	2845	2863	2875	2868.516
Γ_1	2	2	2	2
$\Gamma_{1,1}$	0	30	50	7.057633
A_2	0	1000	2000	542.5347
ω_2	2885	2901	2915	2910.294
Γ_2	2	2	2	2
$\Gamma_{1,2}$	0	10	50	15.0948
A_3	0	500	20000	456.4762
ω_3	2925	2939	2955	2937.486
Γ_3	2	2	2	2
$\Gamma_{1,3}$	0	10	50	7.35773
A_4	0	432	20000	25.24922
ω_4	2950	2967	2980	2966.907
Γ_4	2	2	2	2
$\Gamma_{1,4}$	0	10	50	2.565195

SPP

	Lower Boundary	Initial Conditions	Upper Boundary	Equilibrium
Noise	0	0.0001	2000	3.17E-14
Non Resonant Background	0	50	2000	3.41E+00
A_1	0	1000	2000	1397.664
ω_1	2885	2901	2915	2906.051
Γ_1	2	2	2	2
$\Gamma_{1,1}$	0	10	50	16.61995
A_2	0	500	20000	1.87E+02
ω_2	2930	2947	2960	2942.622
Γ_2	2	2	2	2
$\Gamma_{1,2}$	0	10	50	6.251238

IV. Additional Images

Below are additional images of the same area depicted in the main text with different polarization definition of the NIR, MIR and signal beam lines. The first column for each polarization combination shows the hyperspectral plot generated using the MatLab hyperspectral image processing toolbox. Spectral maps indicate the spectral similarity between pixels by comparing the spectrum of each pixel to the representative spectra identified by the toolbox. The second column depicts the intensity integrated spectra over the entire frequency region of interest. The third column shows the representative spectra of a pixel extracted from the hypercube data set and has the same coloring as that of the first column spectral maps. Different polarization definitions highlight different regions, spectrally and spatially, that exist on the sample area which emphasizes the power of this technique.

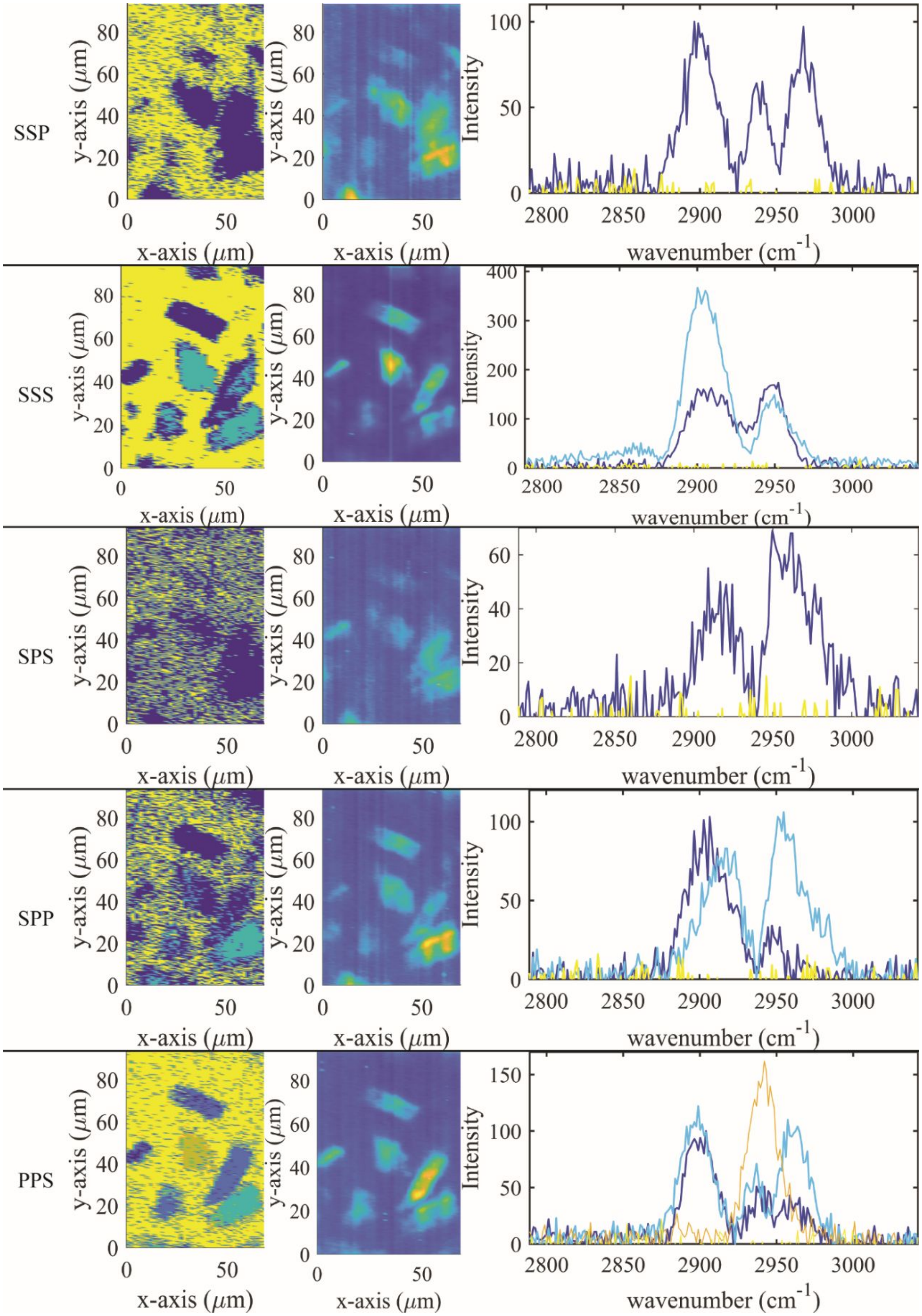

Figure S11 Column 1 shows the spectral map, which is the correlation between endmembers, unique spectra found in the hyperspectral data stack, and spatial coordinates. Column 2 shows the intensity map of the polarization resolved image which is generated by summing all intensity values of a single spatial point. Column 3 shows the representative spectra used to generate

the coloring scheme in the spectral map, which is signal from a single pixel. Integrated spectra over multiple pixels within a sheet are shown in Fig. S9-10, with significantly improved signal-to-noise ratios.

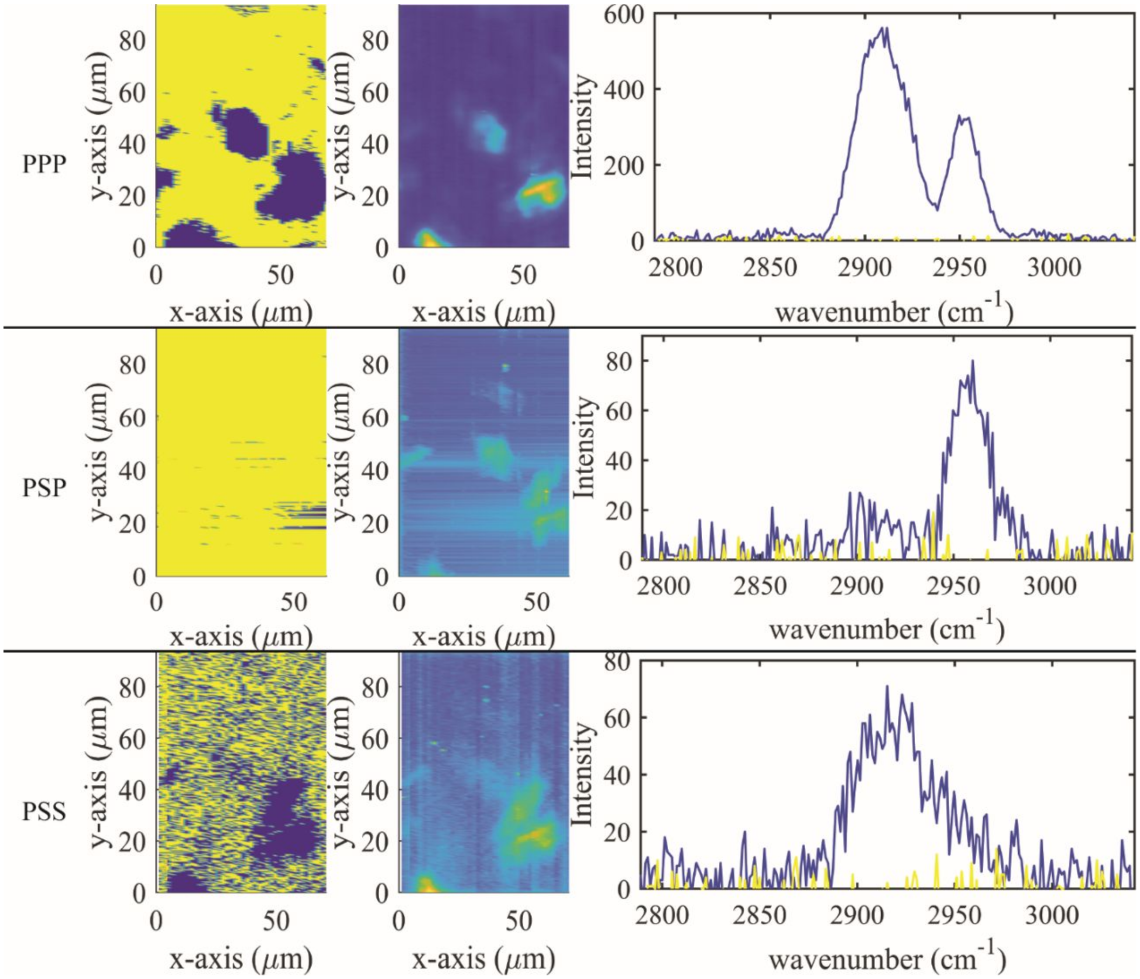

Figure S11 continued

V. Neural Network Solver

As depicted in the workflow in Fig. S13a, a training set with 100,000 data entries was built by first randomly generating $\theta 1 \in [0, \pi)$, $\varphi 1 \in [0, 2\pi)$, $\beta_{xxz} \in (-1, 1)$, $\beta_{zxx} \in (-1, 1)$, $\beta_{xzx} \in (-1, 1)$, $\beta_{xzy} + \beta_{zxy} \in (-2, 2)$, $\beta_{zzz} \in (-1, 1)$, $\beta_{xyz} - \beta_{zxy} \in (-2, 2)$, and $N \in (0, 10)$. $\varphi 2 = \varphi 1 + 60°$. $\theta 2 = \theta 1$ (both sheets facing up) or $\theta 2 = \pi - \theta 1$ (one sheet facing up another down). Here we have 11 terms (9 independent), with $\varphi 2$ and $\theta 2$ to be dependent on $\varphi 1$ and $\theta 1$ respectively and $\beta_{xzy} + \beta_{zxy}$ as well as $\beta_{xyz} - \beta_{zxy}$ to be two grouped terms. Each random generation produced 11 values for (θ,φ,β_{ijk} ,N), and was repeated for 100,000 times to produce 1,100,000 values. Each 11-value subset was then plugged into Eq. 5-12, to calculate 8 $\chi^{(2)}$ values for sheet1 and 8 $\chi^{(2)}$ values for sheet2. In details, ($\theta 1$,$\varphi 1$,β_{xxz},β_{zxx},β_{xzx},β_{zzz}, $\beta_{xzy} + \beta_{zxy}$, $\beta_{xyz} - \beta_{zxy}$) were plugged into Eq. 5-12 for sheet1. Then after plugging in ($\theta 2$,$\varphi 2$,β_{xxz},β_{zxx},β_{xzx}, β_{zzz}, $\beta_{xzy} + \beta_{zxy}$, $\beta_{xyz} - \beta_{zxy}$), the righthand sides of the Eq. 5-12 were multiplied with N, to obtain the 8 $\chi^{(2)}$ values for sheet2. Here we assume sheet1 and sheet2 to have the same β_{ijk} terms as they are formed from chemically identical building blocks SDS@2β-CD and they have the same tilt angle versus surface normal. N was included to scale for surface coverage difference between sheets (*i.e.* sheet1 and sheet2 might have different number of layers/thickness, as shown in the AFM height profile in Fig. S3). With 11 random generated values, we obtain 16 values via Eq. 5-12, together forming a training set with 100,000 entries where each entries have 16+11 values.

To train the neural network, we then used the calculated $\chi^{(2)}$ values (a total of 100,000 by 16 values) as input, and the corresponding 100,000 by 11 (θ,φ,β_{ijk},N) as true values of outputs for the model to learn to predict 11 (θ,φ,β_{ijk},N) values when supplied 16 $\chi^{(2)}$ values. The training is run with an epoch size of 1000 (passing through the entire training set 1000 times) and a batch size of 100 (100 entries as a subset to learn). To avoid overfitting (*i.e.*, neural network model memorizes the training set data well but cannot generalize to data it has not encountered), 90% of the training set (90,000 entries) is used for actual training and 10% is used for test/validation. Fig. S13b shows the model loss during training, calculated by taking the mean squared errors (MSE) between the predicted (θ,φ,β_{ijk},N) vs the true values, for the training and the test set, respectively. It is clear that with more and more learning cycles (*i.e.*, after more epoch runs) the MSE is smaller, indicating less deviation of the prediction from true values. In addition, test set model loss is almost the same as the loss in training set, indicating that even when supplying the model with data not included in the actual training set, it can predict relatively well, and should work for experimental data too.

After the training, we built a new test set with 1000 entries and supply to our trained model for testing. We plot the predicted vs true value to visually inspect the deviation of prediction (Fig. 5b in main text) and calculate the MSE to quantitively analyze it. Fig. 5b in main text plot the predicted value of θ,φ versus their true values in test set, with the MSE to be 3° and 0.4°, respectively. Since θ is < 30° based on the schemetic illustration in Fig. S12, we determine the MSE ° to be 1.5°.

Eventually, by supplying the experimentally retrevied $\chi^{(2)}$ values obtained via spectral fitting (see Eq. 13 and Table S2), we obtained the tilt angle of the SDS@2β-CD subunits to be 23±1.5°. Further discussion and validation can be found in Fig. S4 and S12. In short, from XRD data, it is

very likely that the subunits are tilted to a ~20° level, calculated from the smallest 2theta angle. And using the in-plane unit cell parameters reported by the Jiang group, the subunits cannot lie flat or tilt more than 30°.

We can perform similar error analysis on β_{ijk} and in fact Fig. S13b shows the model loss from the combined MSE of $\theta,\varphi,\beta_{ijk},N$. However, there are no known hyperpolarizability values of SDS@2β-CD for us to further compare and validate. Therefore, we did not perform or demonstrate further analysis on β_{ijk}. Should hyperpolarizability become the knowledge of interests for future studies, it can be extracted using the same method.

When predicting with experimental data, we enumerate the sign of $\chi^{(2)}$ values. With our VSFG as a homodyne technique, i.e., $\chi^{(2)}$ values extracted has no + or − sign representing the phase information, we have to manually enumerate the sign, which is a combination of 2^{16} for 16 $\chi^{(2)}$ values. Moreover, similar to simple trigonometric function where $\theta = 0$ and $\theta = \pi$ both satisty $sin\,(\theta) = 0$, one input vector (16 susceptibility $\chi^{(2)}$ values) in our model could also have multiple output vectors (different in-plane rotation, tilt angle, hyperpolarizabilities combinations) at the same time. Hence, in-plane rotation angle is divided into [0, π) and [π, 2π) intervals and the tilt angle is divided into [0, π/2) and [π/2, π) intervals to differentiate these output vectors. With 2^{16} inputs, we will obtain 2^{16} outputs of $(\theta,\varphi,\beta_{ijk},N)$. We use MSE to score and sort the solutions (Table S2 top workflow chart). In details, with each $(\theta,\varphi,\beta_{ijk},N)$ output, we can also supply them back to Eq. 5-12 to obtain the $\chi^{(2)}$ values. We can then calculate the MSE of the calculated $\chi^{(2)}$ values vs the input sign-enumerated $\chi^{(2)}$ values. Table S2 shows the solution with smallest MSE of $\chi^{(2)}$ of 0.02, where the tilt angle is: 23±1.5°

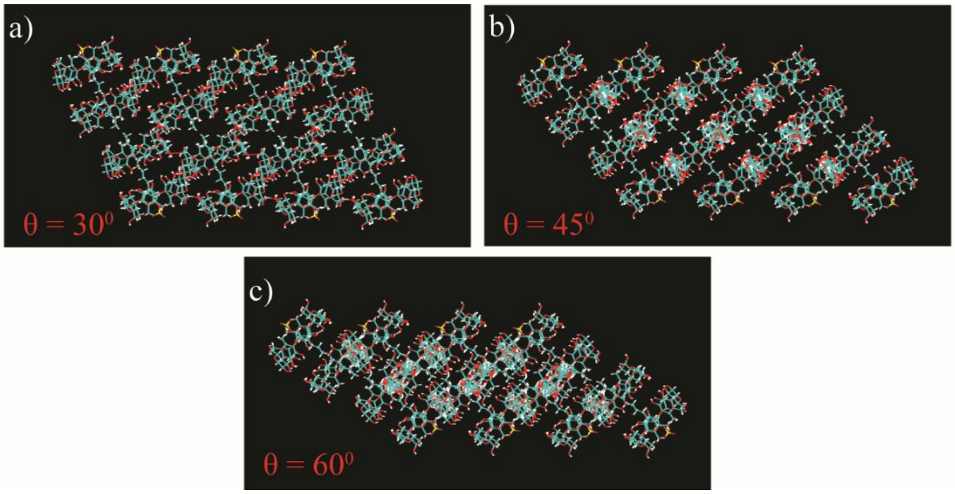

Figure S12 Visualization of the MSA SDS@2β-CD with different degrees of tilting. In-plane unit cell parameters reported by the Jiang group were used.[1]

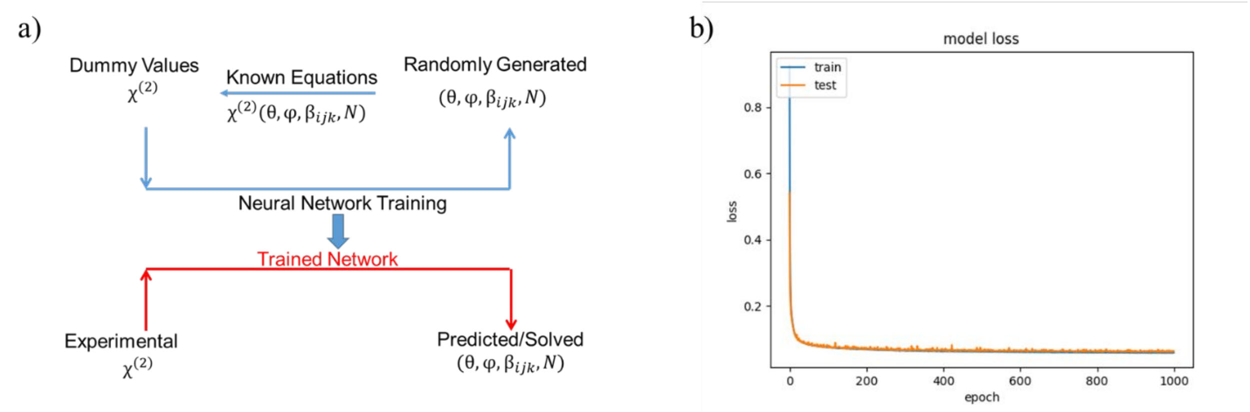

Figure S13 a) Workflow for neural network training and the utilization of trained neural network to extract orientation details. Randomly generated variables $(\theta,\varphi,\beta_{ijk},N)$ were plugged into the set of known equations Eq. 5-12 to calculate the corresponding susceptibility values $\chi^{(2)}$,which were then used back as training set to train the neural network to predict the $(\theta,\varphi,\beta_{ijk},N)$ when supplying $\chi^{(2)}$ values as input. After the model was trained, experimentally determined $\chi^{(2)}$ values were given to the neural network to solve for the molecular-level details. b) Training of the neural network model were run with an epoch size of 1000 and the training loss as well as the validation data set loss (test set) were monitored. Both loss values decreased with more training cycle and eventually reached a plateau, meaning a better fit and prediction were reached. The small loss values indicate a relatively small deviation between the predicted value and true value. Using a validation data set to test the model during the training, we can eliminate the overfitting scenario where the model only memorizes the training data but cannot generalize to unseen one.

Table S2 Susceptibilities extracted from spectral fitting and the corresponding predicted values (normalized).

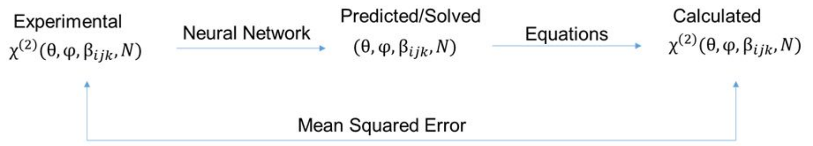

$\chi^{(2)}$ Mean Squared Error: 0.02

	$\chi_{XXX}1$	$\chi_{XXY}1$	$\chi_{XYX}1$	$\chi_{YXX}1$	$\chi_{XYY}1$	$\chi_{YXY}1$	$\chi_{YYX}1$	$\chi_{YYY}1$	$\chi_{XXX}2$	$\chi_{XXY}2$	$\chi_{XYX}2$	$\chi_{YXX}2$	$\chi_{XYY}2$	$\chi_{YXY}2$	$\chi_{YYX}2$	$\chi_{YYY}2$
**Experiment $\chi^{(2)}$ Value**	588	290	62	283	169	327	380	324	1181	369	271	699	0	373	495	213
**Normalized $\chi^{(2)}$ Input**	0.498	-0.246	-0.053	0.240	0.143	0.277	-0.321	-0.275	1.000	0.313	0.230	0.592	0.000	0.316	-0.419	0.181
**Calculated $\chi^{(2)}$**	0.456	-0.296	0.007	0.394	0.099	0.355	-0.283	-0.239	0.683	0.409	0.061	0.634	-0.076	0.553	-0.519	0.473

φ_1	θ_1	β_{zzz}	β_{xxz}	β_{zxx}	β_{xzx}	$\beta_{xzy}+\beta_{zxy}$	$\beta_{xyz}-\beta_{zxy}$	φ_2	θ_2	N
5.82	2.75	-0.28	0.86	0.85	0.54	1.14	-0.43	0.59	0.39	1.59

1 S. Yang, Y. Yan, J. Huang, A. V. Petukhov, L. M. J. Kroon-Batenburg, M. Drechsler, C. Zhou, M. Tu, S. Granick and L. Jiang, *Nat. Commun.*, 2017, **8**, 1–7.
